# Robust Miniemulsion PhotoATRP Driven by Red and Near-Infrared Light

**DOI:** 10.1021/jacs.4c02553

**Published:** 2024-05-01

**Authors:** Xiaolei Hu, Rongguan Yin, Jaepil Jeong, Krzysztof Matyjaszewski

**Affiliations:** Department of Chemistry, Carnegie Mellon University, Pittsburgh, Pennsylvania 15213, United States

## Abstract

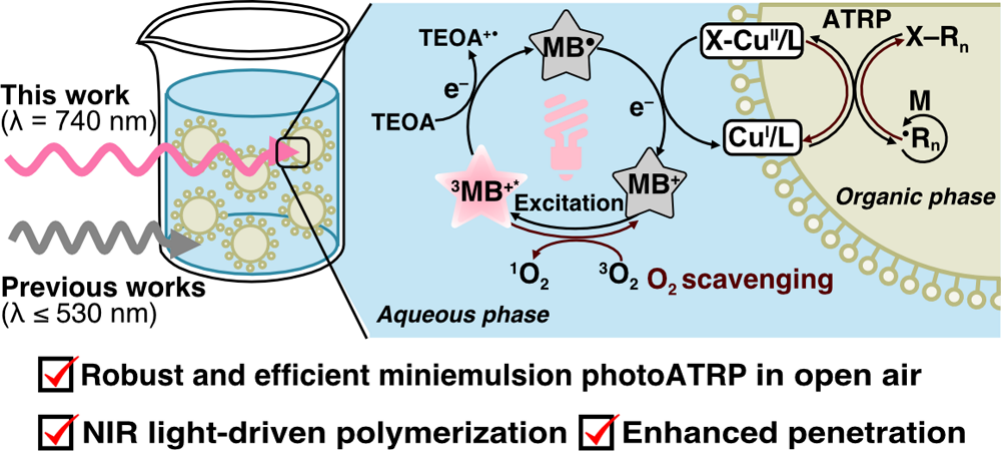

Photoinduced polymerization techniques have gathered significant attention due to their mild conditions, spatiotemporal control, and simple setup. In addition to homogeneous media, efforts have been made to implement photopolymerization in emulsions as a practical and greener process. However, previous photoinduced reversible deactivation radical polymerization (RDRP) in heterogeneous media has relied on short-wavelength lights, which have limited penetration depth, resulting in slow polymerization and relatively poor control. In this study, we demonstrate the first example of a highly efficient photoinduced miniemulsion ATRP in the open air driven by red or near-infrared (NIR) light. This was facilitated by the utilization of a water-soluble photocatalyst, methylene blue (MB^+^). Irradiation by red/NIR light allowed for efficient excitation of MB^+^ and subsequent photoreduction of the ATRP deactivator in the presence of water-soluble electron donors to initiate and mediate the polymerization process. The NIR light-driven miniemulsion photoATRP provided a successful synthesis of polymers with low dispersity (1.09 ≤ *Đ* ≤ 1.29) and quantitative conversion within an hour. This study further explored the impact of light penetration on polymerization kinetics in reactors of varying sizes and a large-scale reaction (250 mL), highlighting the advantages of longer-wavelength light, particularly NIR light, for large-scale polymerization in dispersed media owing to its superior penetration. This work opens new avenues for robust emulsion photopolymerization techniques, offering a greener and more practical approach with improved control and efficiency.

## Introduction

Emulsion polymerization offers a highly advantageous and practical approach to polymer synthesis.^1−8^ The process demonstrates excellent heat transfer, thereby contributing to enhanced energy efficiency and control over polymerization. Additionally, it reduces the amount of toxic and volatile organic solvents, minimizing environmental impact and aligning with eco-friendly practices. The resulting polymer dispersions typically exhibit low viscosity, facilitating handling and formulation in industrial applications. These combined benefits make emulsion polymerization a preferred method, promoting sustainability, safety, and operational efficiency across various industrial processes. The versatility and environmental advantages of emulsion polymerization have led to its widespread adoption in industrial settings for producing polymers used in applications such as coatings, adhesives, and textiles.^9^

Emulsion polymerization has primarily been conducted through a free radical polymerization (FRP) process. However, the intrinsic limitations of FRP, such as uncontrolled molecular weight, broad molecular weight distribution, and limited control over polymer architecture, have been the concern of conventional emulsion polymerizations and their products.^1^ A promising alternative would be adopting reversible deactivation radical polymerization (RDRP) techniques for emulsion polymerization.^4,10−15^ Unlike FRP, propagating radicals in RDRP undergo reversible deactivation mediated by various RDRP-regulating agents (typically the Cu complex for atom transfer radical polymerization (ATRP), chain transfer agents for reversible addition–fragmentation chain transfer (RAFT) polymerization, and alkoxyamine for nitroxide-mediated polymerization (NMP)). This prolongs the lifetime of propagating radicals, compressing undesired chain terminations.^16−21^ Consequently, this affords precise control over molecular weight, dispersity, sequence, end group functionality, and architecture.^22−26^ These advantages have propelled the practical implementation of RDRP in dispersed media.^27,28^ The aqueous dispersed polymerization can generally be categorized as microemulsion, miniemulsion, emulsion, suspension, dispersion, and precipitation polymerization. These different techniques of dispersed polymerization are distinguished based on the initial state of the polymerization mixture, the kinetics of the polymerization process, the mechanism of particle formation, and the size of the resulting particles.^3,4,29^

**Scheme 1:**
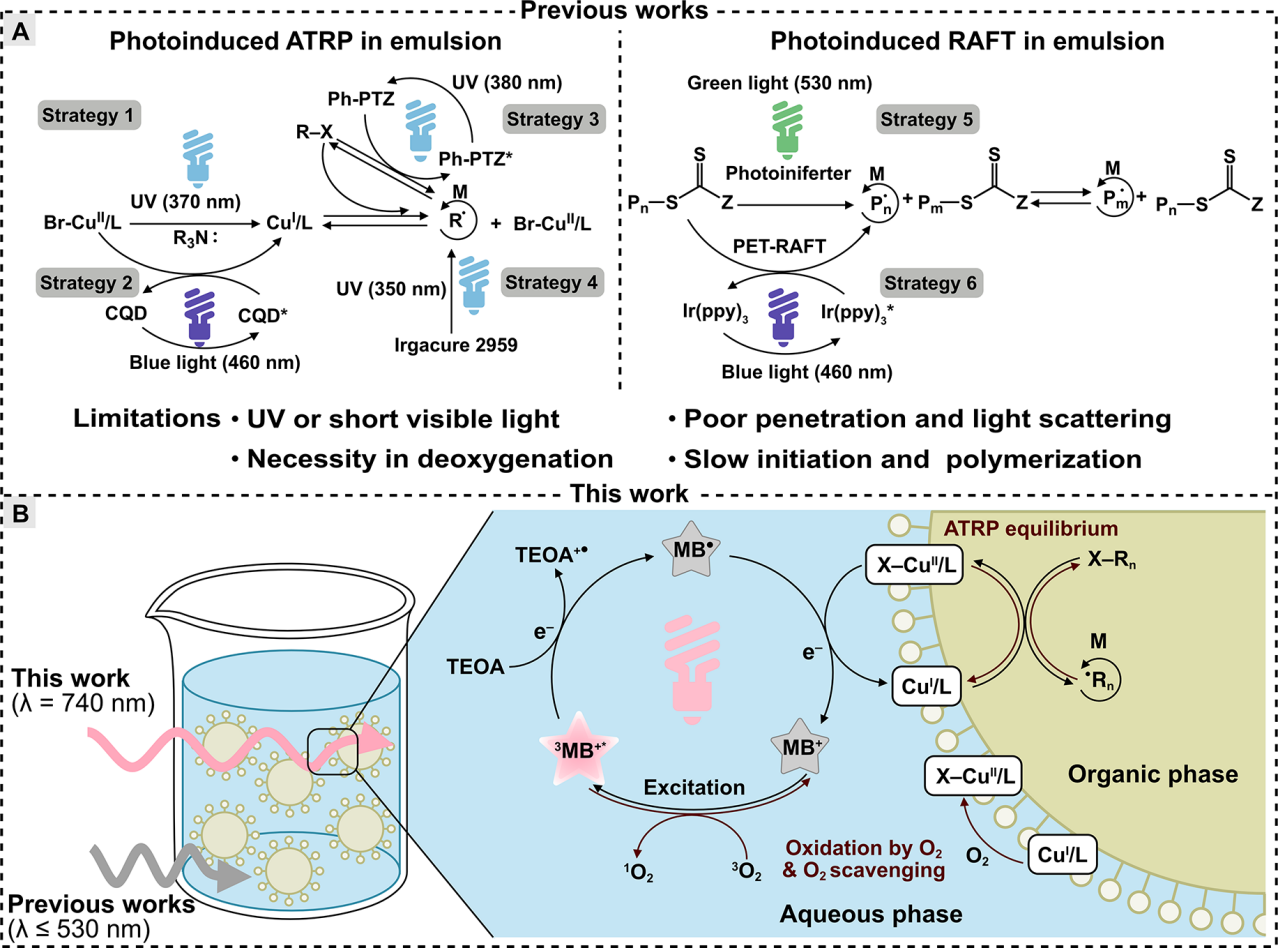
(A) Summary of Previous Works on the Photoinduced RDRP in Dispersed Media; (B) Proposed Mechanism for This Work: Red/NIR Light-Driven Miniemulsion PhotoATRP Mediated by MB^+^ and the Ion-Pair Catalyst [Br–Cu^II^L^+^/DS^–^] at the Interface

Importantly, the RDRP process can be controlled by external stimuli, such as redox reagents (e.g., tin compounds, ascorbic acid),^30^ enzymes,^31,32^ ultrasound,^33,34^ electric current,^35−37^ or light.^38−43^ Among these, light has emerged as a particularly promising approach due to relatively milder reaction conditions, spatiotemporal control, and convenient setup.^44−48^ Previous efforts on the photoinduced RDRP (photoRDRP) in dispersed media include (inverse) microemulsion,^49^ miniemulsion,^50−57^ emulsion,^58−61^ and dispersion (Scheme 1A).^62−64^ For instance, photoATRP in emulsion can be initiated through the in situ generation of the ATRP activator [Cu^I^/L]^+^ (where L is an ATRP ligand). This could be achieved by either UV irradiation (370 nm)^53^ or electron transfer from an excited photocatalyst (PC) under blue light (460 nm),^54^ reducing [X–Cu^II^/L]^+^ (where X = Br or Cl). Alternatively, the ATRP process could be initiated by the direct generation of propagating radicals. This could be achieved by the use of a photoinitiator under UV light^49^ or the direct cleavage of alkyl halide bonds in the presence of photocatalysts such as phenothiazine derivative under UV irradiation.^65,66^ Photoinduced RAFT polymerizations in dispersed media have also been reported. Similar to ATRP, this can be achieved by directly generating propagating radicals through a photoiniferter or a photoinduced electron/energy transfer (PET) process from an excited photosensitizer under UV, blue, or green light (365–530 nm).^50−52^ These previously reported RDRP-based emulsion polymerizations driven by light were capable of polymerization under ambient temperature as well as temporal control.

Nevertheless, all previous reports of photoinduced RDRP in the dispersed medium have relied on UV or short-wavelength visible light (Scheme 1A). Due to the limited penetration and enhanced scattering of short-wavelength lights in heterogeneous systems, polymerizations were often slow, which worsens in large-scale reactions. Consequently, the polymerizations took a long time to achieve high conversion and often showed limited controllability over molecular weights and molecular weight distributions. Moreover, the limited oxygen tolerance of the methods requires rigorous deoxygenation before polymerization, hindering a broader and more practical application of photoinduced RDRP in heterogeneous media.^67,68^ Therefore, the ultimate emulsion photopolymerization technique needs to address the following challenges: (i) the use of long-wavelength light for polymerization; (ii) oxygen tolerance; and (iii) fast polymerization while maintaining good control over the reaction.

To address the challenges and facilitate well-controlled photoRDRP in emulsions, we sought an efficient PC that could be activated under longer wavelength lights, such as red or near-infrared (NIR) lights. Indeed, the superior penetration depth of red/NIR light and their less destructive characteristics have allowed their applications for intracellular polymerization,^69^ protein modification,^70,71^ and polymerization passing through physical barriers.^72−75^ However, many of the previously reported red/NIR light-active PCs exhibited limited solubility in the aqueous phase, which prevented their utilization for polymerization in dispersed media.^72^ This is because the presence of PC in the organic phase (i.e., monomer droplets), instead of the aqueous phase, may deteriorate the photocatalytic activity of PC as a result of solubility change, while the monomer was converted into a polymer.^50^ This suggests that PC should remain in the aqueous phase to maintain its catalytic efficiency.

We envisioned that methylene blue (MB^+^) could be a promising PC for well-controlled photoRDRP in emulsions under red/NIR light. MB^+^ is a commercially available, highly efficient, and water-soluble PC that exhibits photocatalytic activity under a broad range of lights, including red/NIR lights.^76−78^ Our previous study demonstrated that MB^+^ allowed rapid and well-controlled polymerization in homogeneous aqueous phases without deoxygenation.^38^ Motivated by this, we developed a robust photoATRP system for dispersed media that utilizes MB^+^ as the PC under red/NIR light. The schematic illustration of the proposed mechanism is demonstrated in Scheme 1B. First, MB^+^ in the aqueous phase (Figure S2) undergoes excitation by red/NIR lights. The excited triplet state MB^+^ (^3^MB^+^*) is reduced by electron donor (ED) in the aqueous phase to form highly reducing MB radical species (MB^•^), which subsequently reduce [X–Cu^II^/L]^+^ to [Cu^I^/L]^+^ via outer sphere electron transfer.^38^ The use of water-soluble ED (e.g., triethanolamine, TEOA) is crucial for the efficient interaction between ^3^MB^+^* and ED. It also has to be noted that the ATRP catalysts (e.g., Cu^II^/L complex) in dispersed media are predominantly located at the water/organic interphase.^79^ This is because the anionic surfactant, sodium dodecyl sulfate (SDS), binds to the ATRP deactivator [X–Cu^II^/L]^+^ forming [X–Cu^II^L^+^/DS^–^], an interfacial ion-pair catalyst. Therefore, the MB^•^ in the aqueous phase reduces the interfacial ion-pair catalyst [X–Cu^II^L^+^/DS^–^] via electron transfer, generating a [Cu^I^/L]^+^ activator. This induces the rapid initiation of the ATRP process in the organic phase and provides control over polymerization through a reversible redox equilibrium between Cu^I^/Cu^II^ complexes. Additionally, dissolved O_2_ is scavenged by ^3^MB^+^*, MB^•^, or [Cu^I^/L]^+^ species during the dual photocatalytic cycle.^38^ Thus, the NIR-driven miniemulsion photo ATRP could be performed without laborious deoxygenation processes. Consequently, using MB^+^ as a PC offers an efficient and robust route to conducting photoRDRP in dispersed media under red/NIR light, providing excellent oxygen tolerance and precise control.

## Results and Discussion

### Polymerization Conditions

We started with the confirmation of the proposed concept. For the polymerizations, n-butyl methacrylate (BMA) was used as the monomer, ethyl α-bromophenylacetate (EBPA) as the initiator located in the organic phase, [X–Cu^II^/TPMA]^+^ (TPMA = tris(2-pyridylmethyl)amine) complex as the deactivator, MB^+^ as the PC, and TEOA as the ED. SDS was chosen as the surfactant to generate an ion-pair catalyst, together with [X–Cu^II^/TPMA]^+^. Our previous study revealed that the resulting ion-pair catalysts were predominantly (95%) located at the interface of the particles, which facilitated an efficient ATRP process in miniemulsion.^79^ Hexadecane (HD) and water were used as the organic phase and the dispersed medium, respectively. Red LED (640 nm, 25 mW cm^–2^) was initially utilized as the light source for the model reactions. Of note, NaBr was added in the reaction medium to prevent loss of control over the ATRP caused by the dissociation of the [X–Cu^II^/L]^+^ deactivator to the [Cu^II^/L]^2+^ complex, particularly in the presence of anionic surfactant SDS.^80,81^ The miniemulsion photoATRP was performed in a one-dram vial placed in a photoreaction box, without prior deoxygenation (Figures S1 and S2). Monomer conversion was measured by gravimetric analysis.

As shown in entry 1 in Table 1, no polymerization was observed in the absence of PC MB^+^ even after irradiation with red light for 1 h. This indicates that undesired side reactions associated with the direct generation of radicals do not contribute due to the use of long-wavelength light with lower energy. The exclusion of the EBPA initiator (Table 1, entry 2) or ATRP deactivator (i.e., CuBr_2_/TPMA complex (1:1 molar ratio), Table 1, entry 3) led to an uncontrolled free radical polymerization initiated by MB^•^.^38^ In addition, no monomer conversion was observed when polymerization was performed in the absence of the TEOA (Table 1, entry 4) since the generation of MB^•^ was inhibited. In contrast, when all the reagents (i.e., MB^+^, EBPA, CuBr_2_/TPMA, and TEOA) were used together, a rapid and controlled polymerization was observed, achieving a monomer conversion of 48% (*M*_n,abs_ = 15,500, *M*_n,th_ = 13,800, *Đ* = 1.18) within 1 h (Table 1, entry 5). The particle diameter (*Z*_avg_) was 124 nm with a monomodal size distribution of particles (Figures S4), within the range of typical miniemulsion polymerization. These results highlight the critical role of the interfacial catalytic system based on MB^+^/[Br–Cu^II^L^+^/DS^–^] together with water-soluble ED for successful miniemulsion photoATRP under long-wavelength light. Importantly, when hydrophilic ED (i.e., TEOA) was replaced with hydrophobic ED (i.e., excess TPMA), negligible monomer conversion was observed (Table 1, entry 6). This is because hydrophobic TPMA is located in the organic phase (Figure S5) and thus cannot effectively donate an electron to an excited MB^+^, which stays in the aqueous phase. It implies that the use of a water-soluble ED is crucial for photoATRP in dispersed media, unlike conventional photoATRP systems that often use excess TPMA as the ED.^38,41,82^

**Table 1:** Optimization of Polymerization Conditions^a^

entry	CuBr_2_/L (equiv)	TEOA (equiv)	SDS (wt %)	time (h)	conv.^b^ (%)	*M*_n,th_	*M*_n,app_^c^	*M*_n,abs_^d^	*Đ*^c^	*Z*_avg_ (nm)^e^
1^f^	0.1	0.6	4.6	1	1					
2^g^	0.1	0.6	4.6	1	43		481,800	657,900	2.96	76 ± 0.5
3		0.6	4.6	1	95	27,200	174,100	227,800	2.42	77 ± 0.3
4	0.1		4.6	1	0					
5	0.1	0.6	4.6	1	48	13,900	13,200	15,500	1.18	124 ± 0.4
6^h^	0.1	0.6	4.6	1	2					
7	0.025	0.6	4.6	1	77	22,100	26,400	31,900	1.19	99 ± 0.5
8	0.05	0.6	4.6	1	49	14,200	16,700	19,800	1.17	115 ± 0.9
9	0.2	0.6	4.6	2	44	12,700	11,200	13,100	1.14	135 ± 0.7
10	0.3	0.6	4.6	4.5	48	13,900	15,600	18,500	1.09	147 ± 0.9
11	0.1	0.3	4.6	2	48	13,900	13,600	16,000	1.15	114 ± 0.5
12	0.1	0.6	4.6	2	73	21,100	25,200	30,400	1.16	118 ± 1.3
13	0.1	0.9	4.6	2	97	27,800	31,900	38,900	1.47	118 ± 0.7
14	0.1	1.2	4.6	2	99	28,400	35,400	43,400	1.71	123 ± 0.8
15	0.1	0.6	6.9	2	98	28,100	30,500	37,100	1.27	109 ± 0.7
16	0.1	0.6	9.2	1	89	25,500	26,800	32,400	1.19	82 ± 0.5
17^i^	0.1	0.6	9.2	1	99	28,400	25,900	31,300	1.31	84 ± 0.5

a Reaction conditions: [BMA]/[EBPA]/[MB^+^]/[CuBr_2_/TPMA (1:1 molar ratio)]/[TEOA] = 200/1/0.025/*X*/*Y*, [M] = 20 vol % to total, [HD] = 10.8 wt % to BMA, [SDS] = *Z* wt % relative to BMA, [NaBr] = 0.1 M, irradiated under red LED (640 nm, 25 mW cm^–2^) in a one-dram vial (diameter = 15 mm) with stirring, in open air.

b Monomer conversion was determined by gravimetric analysis.

c Molecular weight (*M*_n,app_) and dispersity (*Đ*) were determined by SEC analysis (THF as the eluent) calibrated to polystyrene standards.

d Absolute molecular weight (*M*_n,abs_) was determined by Mark–Houwink calibration.^83,84^

e Average particle diameter (*Z*_avg_) was determined by DLS.

f No MB^+^.

g No EBPA.

h TPMA was used instead of TEOA.

i Less HD (3 wt %) was used.

Further investigation was performed to examine the effect of the ATRP components on the polymerization by adjusting the concentration of the ATRP deactivator, [Br–Cu^II^/TPMA]^+^. Similar to ATRP in homogeneous systems, higher concentrations of [Br–Cu^II^/TPMA]^+^ allowed better control over polymerization. To be specific, lower dispersity values of up to 1.09 were observed when the deactivator concentration was increased (Table 1, entries 5, 7–10, Figures S6–S8). The effect of ED on polymerization performance was also investigated. Using TEOA at 0.3 equiv (with respect to EBPA), the polymerization proceeded moderately with 48% monomer conversion achieved after 2 h (Table 1, entry 11). By increasing the TEOA concentration by 1- and 2-fold (0.6 and 0.9 equiv), the monomer conversion significantly increased to 73 and 97%, respectively (Table 1, entries 12 and 13), due to more efficient electron transfer from TEOA to ^3^MB^+^*. Interestingly, a further increase in TEOA concentration (1.2 equiv) resulted in a higher dispersity of 1.71 (Table 1, entry 14). This could be attributed to the overreduction of Cu^II^ to the Cu^I^ complex, caused by rapid electron transfer in the presence of a large excess of TEOA, which led to the loss of control. We further investigated the effect of the concentration of the surfactant SDS. When the concentration of SDS was increased from 4.6 (Table 1, entry 12) to 6.9 and 9.2 wt % relative to monomer (Table 1, entries 15 and 16), the *Z*_avg_ of the resulting particle decreased from 118 to 82 nm, as observed in a previous emulsion system.^53^ Higher SDS concentrations also led to an increased polymerization rate. This effect was attributed to smaller particle size, reduced light scattering, improved light penetration, and diminished radical termination due to enhanced compartmentalization effects.^53,54,85^ Additionally, higher SDS concentration should lead to more particle formation during the nucleation stage and a larger total surface area of resulting particles, thereby facilitating more effective interfacial catalysis mediated by the ion-pair catalyst in miniemulsion ATRP.^86^ These results imply that modulating the SDS concentration could be another route to controlling the particle size and polymerization rate. Finally, the impact of the costabilizer HD on polymerization was explored. The results indicated rapid polymerization even when the HD concentration was reduced from 10.8 to 3 wt % relative to the monomer (Table 1, entry 17). However, an increased dispersity was observed with *Đ* increasing from 1.19 to 1.31. This is likely due to less effective compartmentalization and relatively poor colloidal stability at a lower amount of HD. DLS results revealed a bimodal size distribution of the particles at a lower HD of 3 wt % (Figure S4, entry 17).

### Mechanistic Discussion

The results presented in Table 1 suggest that miniemulsion photopolymerization mediated by MB^+^ follows a similar mechanism established in photoATRP under homogeneous conditions.^38^ The water-soluble TEOA (*E*_1/2_(TEOA^+•^/TEOA) = +0.7 V vs SCE)^87^ plays a crucial role as a sacrificial electron donor which reductively quenches ^3^MB^+^* (*E*_1/2_(^3^MB^+^*/MB^•^) = +1.60 V vs SCE) in the aqueous phase (Scheme 1B). This results in the formation of the semireduced MB radical (MB^•^) and an amine radical cation (TEOA^+•^). Subsequently, MB^•^ (*E*_1/2_ (MB/MB^•^) = −0.30 V vs SCE) reduces [Br–Cu^II^L^+^/DS^–^] (*E*_1/2_(Cu^II^/Cu^I^) = −0.23 V vs SCE) at the water/oil interface by single electron transfer, generating the [Cu^I^L^+^/DS^–^] complex and reforming the MB^+^ in the ground state. The resulting [Cu^I^L^+^/DS^–^] initiates polymerization and controls radical propagation in the organic phase by a reversible redox equilibrium between Cu^I^/Cu^II^ complexes, where they intermittently activate dormant species and deactivate radicals. In summary, water-soluble photocatalyst MB^+^ and electron donor TEOA, together with the interfacial ion-pair catalyst [Br–Cu^II^L^+^/DS^–^], are essential for the induction and sustainment of red light-mediated miniemulsion ATRP.

### Kinetic Study

**Figure 1:**
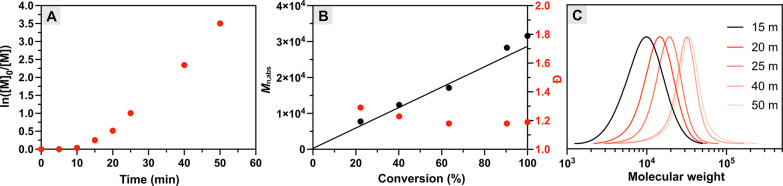
Miniemulsion photoATRP under NIR light. (A) First-order kinetic plot. (B) Evolution of molecular weights and molecular weight distributions with monomer conversion. (C) SEC traces evolution over time. Reaction conditions: [BMA]/[EBPA]/[MB^+^]/[CuBr_2_/TPMA]/[TEOA] = 200/1/0.025/0.1/0.6, [M] = 20 vol % to total, [HD] = 10.8 wt % to BMA, [SDS] = 9.2 wt % relative to BMA, [NaBr] = 0.1M, irradiated under NIR LED (740 nm, 20 mW cm^–2^) in a one-dram vial (diameter = 15 mm) with stirring, in open air. Monomer conversion was determined by gravimetric analysis. Molecular weight (*M*_n,app_) and dispersity (*Đ*) were determined by SEC analysis (THF as the eluent) calibrated to polystyrene standards. Absolute molecular weight (*M*_n,abs_) was determined by Mark–Houwink calibration.

A previous study on the photoATRP using MB^+^ in a homogeneous system revealed that MB^+^ can mediate photopolymerization under a broad range of lights from UV to NIR light.^38^ Inspired by this, we investigated the capability of the miniemulsion photoATRP system to proceed under NIR light (740 nm), beyond red light. The reaction condition for entry 16 in Table 1 was selected as the standard polymerization condition because it exhibited a high conversion of 89% and a decent dispersity of 1.19 reached within 1 h. The kinetic analysis under NIR light irradiation revealed a short induction period of ca. 10 min, accounting for the consumption of O_2_ in the reaction mixture, followed by rapid polymerization, achieving a nearly quantitative monomer conversion within 50 min (Figure 1A). The absolute molecular weights (*M*_n,abs_) increased linearly as a function of monomer conversion, while a narrow molecular weight distribution was maintained (1.15 ≤ *Đ* ≤ 1.29, Figure 1B). Also, *M*_n,abs_ was in good agreement with theoretical values (*M*_n,th_, solid line in Figure 1B). In addition, the monomodal SEC traces shifted toward the high molecular weight (MW) region with a prolonged irradiation time (Figure 1C). We also tested NIR light with a longer wavelength (808 nm) for the miniemulsion photoATRP; however, no monomer conversion was observed within 1 h, likely due to the negligible light absorption of methylene blue in that region.^38^ These results demonstrated that successful and efficient miniemulsion photoATRP could be achieved under NIR light (740 nm) beyond red light. We also investigated the kinetics of miniemulsion photoATRP using other lights, including UV (Figure S9), green (Figure S10), and red lights (Figure S11), respectively. Although first-order kinetic plots were observed in all three cases, polymerizations using those lights were significantly slower compared with those under NIR light. This is because the penetration of short-wavelength light in dispersed media was less efficient, which hindered the successful activation of MB^+^ and subsequent initiation and propagation. This is further evidenced by the significant deviation between *M*_n,abs_ and *M*_n,th_ of the polymers synthesized under the shorter-wavelength light (Figures S9B, S10B, S11B). These results demonstrate that the use of longer-wavelength light, such as NIR, is particularly important for efficient and well-controlled photopolymerization in dispersed media.

### Comparison of the Polymerization Efficiency in Homogeneous and Heterogeneous Medium

**Figure 2:**
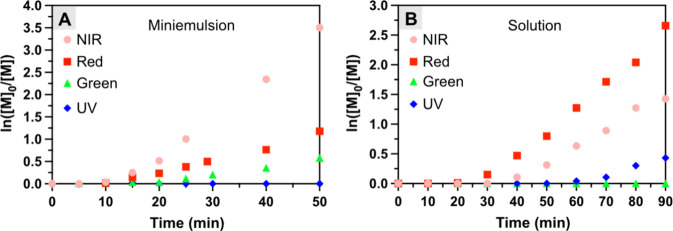
Polymerization kinetics under different light wavelengths in the (A) miniemulsion and (B) homogeneous aqueous phase, respectively. Figure 2A is reproduced from the results shown in Figures 1A and S9A–S11A. Reaction conditions for (A) [BMA]/[EBPA]/[MB^+^]/[CuBr_2_/TPMA]/[TEOA] = 200/1/0.025/0.1/0.6, [M] = 20 vol % to total, [HD] = 10.8 wt % to BMA, [SDS] = 9.2 wt % relative to BMA, and [NaBr] = 0.1 M. Reaction conditions for (B) [OEOMA_500_]/[HO-EBiB]/[MB^+^]/[CuBr_2_]/[TPMA] = 200/1/0.025/0.3/0.9, [OEOMA_500_] = 300 mM, in 1× phosphate buffer solution (PBS) with DMSO (10% v/v). Polymerizations were performed in a one-dram vial (diameter = 15 mm) with stirring in open air.

For a more comprehensive understanding of the miniemulsion photoATRP system, we conducted polymerization in homogeneous aqueous media and compared the polymerization efficiency in homogeneous and heterogeneous conditions. For the polymerization in the homogeneous phase, oligo(ethylene oxide) methyl ether methacrylate (OEOMA_500_, average *M*_n_ = 500) was utilized as a model monomer in water, 2-hydroxyethyl α-bromoisobutyrate (HO-EBiB) as the water-soluble initiator, and MB^+^ as the PC while maintaining the identical photoreaction setup (Figures S1 and S12). Similar to the results from miniemulsion (Figure 2A), linear semilogarithmic kinetic plots were observed for polymerization in a homogeneous aqueous medium under different light sources (Figure 2B). However, under homogeneous conditions, polymerization efficiency followed a different order: red light was the most efficient, followed by NIR, UV, and green light (Figures 2B and S13). This order mostly follows the absorptivity of MB^+^ except NIR.^88^ Despite the relatively lower absorptivity of MB^+^ in the NIR light range compared to green and UV lights, NIR light enabled a faster polymerization rate due to its great penetration. In contrast, the order differed for photopolymerization in miniemulsion and the polymerization rate was positively correlated to the wavelength of light: NIR was the most efficient, followed by red, green, and UV light (Figure 2A and Table 2). This remarkable difference implies that particularly for miniemulsion polymerization, the use of longer-wavelength light and efficient light penetration could be crucial in addition to the absorbance maxima of the PC. This also highlights the significance and importance of our NIR-driven miniemulsion photoATRP system, aligning with previously reported photo-PISA by RAFT polymerization under NIR light.^73^

**Table 2:** Miniemulsion photoATRP of BMA using Different Wavelength Lights^a^

entry	light	λ_max_ (nm)	intensity (mW cm^–2^)	time (min)	conv. (%)^b^	*M*_n,th_	*M*_n,app_^c^	*M*_n,abs_^d^	*Đ*^c^
1	NIR	740	20	40	91	26,000	23,500	28,300	1.18
2	Red	640	25	40	63	18,300	18,600	22,200	1.25
3	Green	520	25	40	30	8,800	8,400	9,700	1.28
4	UV	390	25	40	0				
5	UV	390	25	180	42	12,200	11,400	13,300	1.27

a Reaction conditions: [BMA]/[EBPA]/[MB^+^]/[CuBr_2_/TPMA]/[TEOA] = 200/1/0.025/0.1/0.6, [M] = 20 vol % to total, [HD] = 10.8 wt % to BMA, [SDS] = 9.2 wt % relative to BMA, [NaBr] = 0.1 M, irradiated under different light wavelengths in a one-dram vial (diameter = 15 mm) with stirring, in open air.

b Monomer conversion was determined by gravimetric analysis.

c Molecular weight (*M*_n,app_) and dispersity (*Đ*) were determined by SEC analysis (THF as the eluent) calibrated to polystyrene standards.

d Absolute molecular weight (*M*_n,abs_) was determined by Mark–Houwink calibration.

### Chain Extension

**Figure 3:**
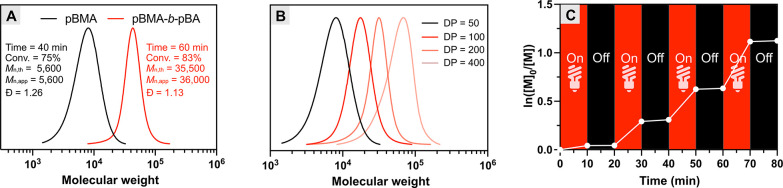
(A) Chain extension of the pBMA macroinitiator with BA. (B) SEC traces of pBMA with different targeted degrees of polymerization. (C) Temporal control of miniemulsion photoATRP under NIR light.

The chain-end fidelity of the polymers synthesized by miniemulsion photoATRP driven by NIR light was examined by chain extension experiments (Figure 3A). First, pBMA was synthesized (*M*_n,app_ = 5,600, *Đ* = 1.26) by miniemulsion photoATRP and then used as a macroinitiator for chain extension with *n*-butyl acrylate (BA). The resulting block copolymer (pBMA-*b*-pBA) showed a low dispersity of 1.13 and a molecular weight (*M*_n,app_) of 36,000, which was in good agreement with the theoretical value (*M*_n,th_ = 35,500). Moreover, the SEC trace of the block copolymer shifted to the higher MW region without tailing or a shoulder peak (Figure 3A). A similar phenomenon (Figure S14) was observed when pBMA was chain extended with BMA, yielding pBMA-*b*-pBMA (*M*_n,app_ = 40,400, *Đ* = 1.26). These results confirm that the NIR-driven miniemulsion photoATRP occurs in a controlled manner, effectively suppressing undesired terminations of polymer chains.

### Tailoring Degrees of Polymerization

Next, we proceeded to examine the capability of miniemulsion photoATRP under NIR light to control the molecular weights of the resulting polymers. By variation of the initiator concentration, while keeping the other polymerization components at constant concentrations, various degrees of polymerization (DP_T_) from 50 to 400 were targeted (Figure 3B, Table S1). The monomer conversion reached 75–99% within 40 min in all cases (Table S1). In addition, the molecular weights (*M*_n,abs_) of the resulting polymers showed good agreement with theoretical values with low dispersity (1.17 ≤ *Đ* ≤ 1.26). This demonstrates the capability of our miniemulsion photoATRP technique to control the molecular weight of the resulting polymer.

### Temporal Control

Temporal control over the polymerization allows for controlling the heat transfer which could be a critical consideration for large-scale emulsion polymerization in industrial settings.^47^ Taking advantage of the photoinduced polymerization, facile temporal control was achieved by switching the light on and off (Figure 3C). Notably, polymerization proceeded exclusively under the irradiation with NIR light. When the light was off, negligible monomer conversion was observed (Table S2). In contrast, polymerization resumed upon the irradiation of NIR light, facilitated by the photoinduced regeneration of the ATRP activator by excited MB^+^. The alternating cycles of NIR light on and off were repeated several times, demonstrating excellent temporal controllability. The resulting polymer showed a good agreement between *M*_n,abs_ and the theoretical value (*M*_n,abs_ = 21,200, *M*_n,th_ = 19,400) with low dispersity (*Đ* = 1.18). Good temporal control was also achieved under red light (Figure S15, Table S3).

### Polymerization Using Different Reactor Sizes

One of the fundamental challenges of previous emulsion photoRDRP originated in using short-wavelength light, which led to light scattering and poor light penetration. This resulted in not only slow initiation and propagation but also side reactions caused by the high energy of short-wavelength lights which often compromised the control of RDRP processes.^47,89^ In fact, a previous study revealed that these concerns become more severe as the size of the reactor for emulsion photopolymerization increases, whereas they are not as severe for a homogeneous polymerization system.^53^ We envisioned that using NIR light could address the scalability-related concerns of photopolymerization in dispersed media. Therefore, inspired by the successful miniemulsion photoATRP under NIR light, we investigated the effect of the size of the reactor on polymerization.

**Figure 4:**
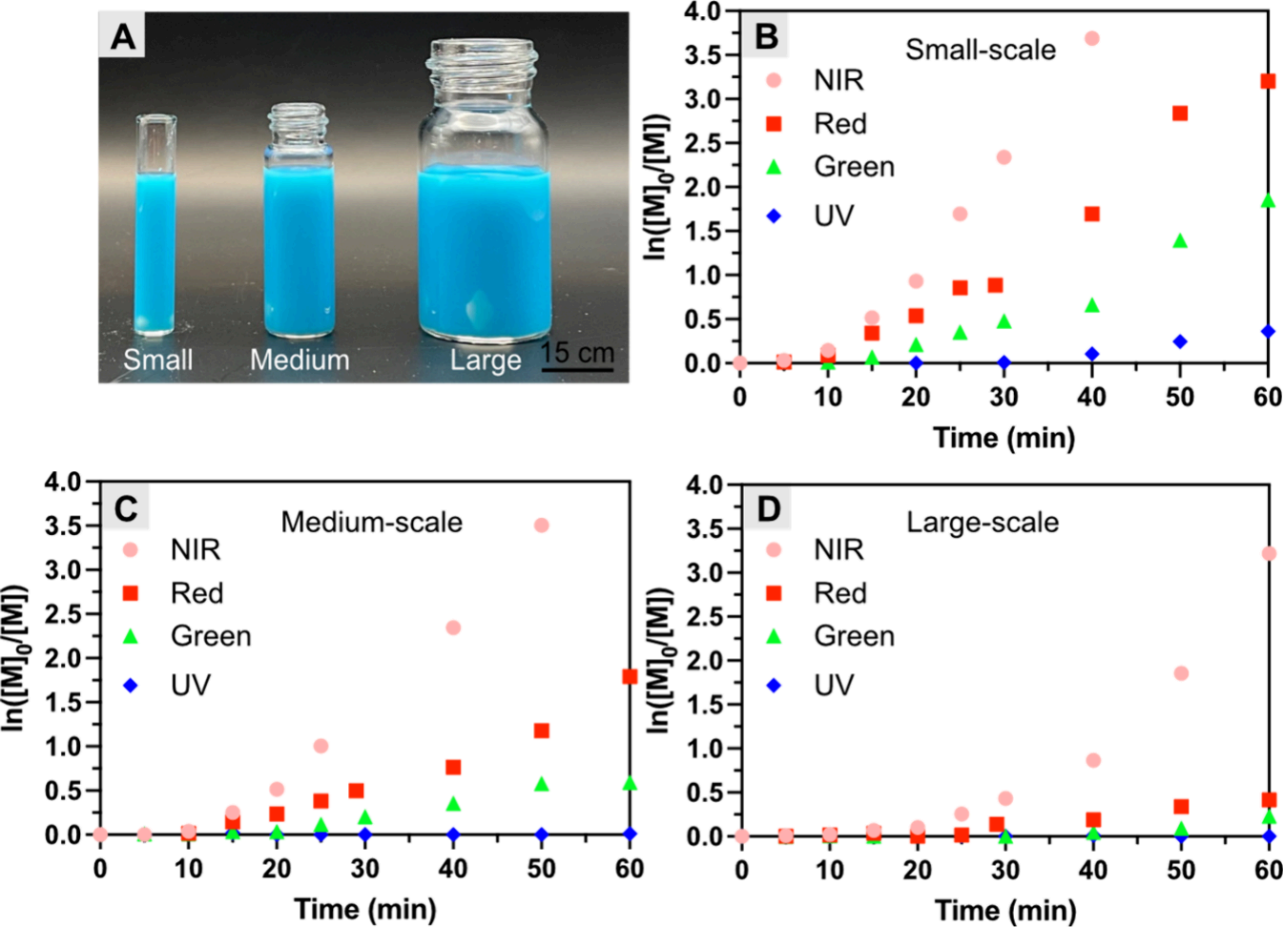
(A) Digital camera image of different reaction vials for miniemulsion photoATRP and kinetics in reactors with the diameters of (B) 7.5 mm; (C) 15 mm; and (D) 27 mm under different light wavelengths, respectively. Reaction conditions: [BMA]/[EBPA]/[MB^+^]/[CuBr_2_/TPMA]/[TEOA] = 200/1/0.025/0.1/0.6, [M] = 20 vol % to total, [HD] = 10.8 wt % to BMA, [SDS] = 9.2 wt % relative to BMA, [NaBr] = 0.1 M, irradiated under different light wavelengths with stirring, in open air.

We performed polymerizations in smaller (diameter = 7.5 mm, abbreviated as S) and larger (diameter = 27 mm, abbreviated as L) glass vials, as compared to the original reactor (diameter = 15 mm, abbreviated as M) utilized for our benchmark reactions (Figure 4). Although no polymerization on the L-scale under UV light was observed (Figure 4D), all other polymerizations showed linear first-order kinetic plots (Figures S16–S22), indicating excellent photocatalytic activity of MB^+^ under different wavelengths. However, near-quantitative conversion, rapid polymerization with a shorter induction period, and low dispersity were only observed under NIR light, regardless of the reactor size (Figure 4, Tables 3 and S4). To be specific, a high conversion of 96% was achieved after polymerization for 1 h under NIR in an L-scale reactor (Table 3, entry 1), accounting for only a 3% decrease in conversion while switching from small to large reactors for polymerization. In contrast, for short-wavelength lights, the conversion significantly decreased when the larger reactor was used for the polymerization. For instance, when the diameter of the reactor was increased from 7.5 to 27 mm, approximately 65, 76, and 100% decreases in monomer conversions were observed for miniemulsion photopolymerization using red, green, and UV lights, respectively (Table 3, entries 2–4). Furthermore, the greater penetration of NIR light drove efficient polymerization through an A4 paper with only a 40% decrease in conversion, as compared to the 63% drop observed for the red light (Figure S23, Table S5).

**Table 3:** Miniemulsion photoATRP of BMA in the Vials with Varying Diameters Using Different Light Wavelengths^a^

entry	light	conversion (%)			conversion drop (small vs large)
		small (7.5 mm)	medium (15 mm)	large (27 mm)	
1	NIR	>99	>99	96	3%
2	Red	96	89	34	65%
3	Green	84	45	20	76%
4	UV	30	1	0	100%

a Reaction conditions: [BMA]/[EBPA]/[MB^+^]/[CuBr_2_/TPMA]/[TEOA] = 200/1/0.025/0.1/0.6, [M] = 20 vol % to total, [HD] = 10.8 wt % to BMA, [SDS] = 9.2 wt % relative to BMA, [NaBr] = 0.1 M, irradiated under different light sources for 1 h with stirring, in open air.

To demonstrate the scalability, we conducted a large-scale reaction with a total volume of 250 mL under NIR light without prior deoxygenation (Figures S24 and S25, Table S6). Quantitative monomer conversion (>99%) was achieved within 3 h with a good correlation of MW with theoretical value (*M*_n,abs_ = 30,800, *M*_n,th_ = 28,400) and low dispersity (*Đ* = 1.26). The resulting particles had a diameter of 107 nm without coagulation. These results, along with the scale-up in the one-dram vial, further highlight that the use of NIR light is highly advantageous, particularly for large-scale polymerization in dispersed media owing to its superior penetration.

## Conclusions

In conclusion, we demonstrated the first example of robust and efficient miniemulsion photoATRP under long-wavelength light, particularly red and NIR lights. This discovery was facilitated by interfacial photocatalysis using MB^+^ as the PC and the interfacial ion-pair catalyst [X–Cu^II^L^+^/DS^–^] for mediating controlled polymerization. Optimization of reaction conditions, including concentrations of the ATRP deactivator, ED, SDS, and HD, identified important parameters for synthesizing polymers with low dispersity and quantitative monomer conversion within 1 h. The proposed mechanism involves the reductive quenching of ^3^MB^+^* by the water-soluble ED (i.e., TEOA), forming MB^•^. Subsequently, MB^•^ reduced [Br–Cu^II^L^+^/DS^–^] at the water/oil interface, generating the [Cu^I^L^+^/DS^–^] complex. The resulting [Cu^I^L^+^/DS^–^] initiated polymerization and controlled radical propagation in the organic phase by a reversible redox equilibrium between the Cu^I^/Cu^II^ complexes. NIR light exhibited superior performance in dispersed media, achieving faster polymerization even at lower intensity compared to other wavelengths, which highlights the importance of adopting longer-wavelength lights. The kinetic study and chain extension experiments confirmed the well-controlled polymerization system. The method allowed for achieving varying degrees of polymerization with high control, showcasing the versatility of the technique. Temporal control experiments demonstrated the ability to switch polymerization on and off in the presence or absence of light, providing a practical route for regulating heat transfer in large-scale applications. Most importantly, the impact of light penetration on polymerization in reactors of different sizes revealed that NIR light maintained efficient polymerization even in larger reactors (250 mL) due to its superior light penetration capabilities. This finding is crucial for the practical application of photoinduced emulsion polymerization. Overall, the NIR light-driven miniemulsion ATRP using MB^+^ offers a promising approach for achieving rapid, controlled, and oxygen-tolerant polymerization in dispersed media. The versatility, efficiency, and potential for temporal control of our methodology make it a valuable contribution to the field of emulsion polymerization, which is anticipated to be extended to other emulsion techniques. Further exploration and application of this technique in industrial settings could lead to the sustainable and economic production of polymers.^89^

## Supporting Information Available

Additional details of experimental procedures and experimental results, including digital camera images of reaction setup, additional SEC traces, kinetic plots, temporal control results, and DLS results (PDF)

## References

[ref1] Lovell P. A.; Schork F. J. Fundamentals of Emulsion Polymerization. Biomacromolecules 2020, 21 (11), 4396–4441. 10.1021/acs.biomac.0c00769. 32543173

[ref2] Chern C. S. Emulsion polymerization mechanisms and kinetics. Prog. Polym. Sci. 2006, 31 (5), 443–486. 10.1016/j.progpolymsci.2006.02.001.

[ref3] Zetterlund P. B.; Kagawa Y.; Okubo M. Controlled/Living Radical Polymerization in Dispersed Systems. Chem. Rev. 2008, 108 (9), 3747–3794. 10.1021/cr800242x. 18729519

[ref4] Zetterlund P. B.; Thickett S. C.; Perrier S.; Bourgeat-Lami E.; Lansalot M. Controlled/Living Radical Polymerization in Dispersed Systems: An Update. Chem. Rev. 2015, 115 (18), 9745–9800. 10.1021/cr500625k. 26313922

[ref5] Cunningham M. F. Living/controlled radical polymerizations in dispersed phase systems. Prog. Polym. Sci. 2002, 27 (6), 1039–1067. 10.1016/S0079-6700(02)00008-4.

[ref6] Cunningham M. F. Controlled/living radical polymerization in aqueous dispersed systems. Prog. Polym. Sci. 2008, 33 (4), 365–398. 10.1016/j.progpolymsci.2007.11.002.

[ref7] Min K.; Matyjaszewski K. Atom transfer radical polymerization in aqueous dispersed media. Cent. Eur. J. Chem. 2009, 7 (4), 657–674. 10.2478/s11532-009-0092-1.

[ref8] Wang Y.; Lorandi F.; Fantin M.; Matyjaszewski K. Atom transfer radical polymerization in dispersed media with low-ppm catalyst loading. Polymer 2023, 275, 125913 10.1016/j.polymer.2023.125913.

[ref9] Chern C.-S. Principles and applications of emulsion polymerization; John Wiley & Sons, 2008.

[ref10] Qiu J.; Gaynor S. G.; Matyjaszewski K. Emulsion Polymerization of n-Butyl Methacrylate by Reverse Atom Transfer Radical Polymerization. Macromolecules 1999, 32 (9), 2872–2875. 10.1021/ma981695f.

[ref11] Liu D.; He J.; Zhang L.; Tan J. 100th Anniversary of Macromolecular Science Viewpoint: Heterogenous Reversible Deactivation Radical Polymerization at Room Temperature Recent Advances and Future Opportunities. ACS Macro Lett. 2019, 8 (12), 1660–1669. 10.1021/acsmacrolett.9b00870. 35619385

[ref12] Monteiro M. J.; Cunningham M. F. Polymer Nanoparticles via Living Radical Polymerization in Aqueous Dispersions: Design and Applications. Macromolecules 2012, 45 (12), 4939–4957. 10.1021/ma300170c.

[ref13] Clothier G. K. K.; Guimarães T. R.; Thompson S. W.; Rho J. Y.; Perrier S.; Moad G.; Zetterlund P. B. Multiblock copolymer synthesis via RAFT emulsion polymerization. Chem. Soc. Rev. 2023, 52 (10), 3438–3469. 10.1039/D2CS00115B. 37093560

[ref14] Prescott S. W.; Ballard M. J.; Rizzardo E.; Gilbert R. G. RAFT in Emulsion Polymerization: What Makes it Different?. Aust. J. Chem. 2002, 55 (7), 415–424. 10.1071/CH02073.

[ref15] Gaynor S. G.; Qiu J.; Matyjaszewski K. Controlled/“Living” Radical Polymerization Applied to Water-Borne Systems. Macromolecules 1998, 31 (17), 5951–5954. 10.1021/ma980724j. 9680462

[ref16] Corrigan N.; Jung K.; Moad G.; Hawker C. J.; Matyjaszewski K.; Boyer C. Reversible-deactivation radical polymerization (Controlled/living radical polymerization): From discovery to materials design and applications. Prog. Polym. Sci. 2020, 111, 101311 10.1016/j.progpolymsci.2020.101311.

[ref17] Parkatzidis K.; Wang H. S.; Truong N. P.; Anastasaki A. Recent Developments and Future Challenges in Controlled Radical Polymerization: A 2020 Update. Chem. 2020, 6 (7), 1575–1588. 10.1016/j.chempr.2020.06.014.

[ref18] Hill M. R.; Carmean R. N.; Sumerlin B. S. Expanding the Scope of RAFT Polymerization: Recent Advances and New Horizons. Macromolecules 2015, 48 (16), 5459–5469. 10.1021/acs.macromol.5b00342.

[ref19] Matyjaszewski K.; Xia J. Atom Transfer Radical Polymerization. Chem. Rev. 2001, 101 (9), 2921–2990. 10.1021/cr940534g. 11749397

[ref20] Matyjaszewski K. Atom Transfer Radical Polymerization (ATRP): Current Status and Future Perspectives. Macromolecules 2012, 45 (10), 4015–4039. 10.1021/ma3001719.

[ref21] Hawker C. J.; Bosman A. W.; Harth E. New Polymer Synthesis by Nitroxide Mediated Living Radical Polymerizations. Chem. Rev. 2001, 101 (12), 3661–3688. 10.1021/cr990119u. 11740918

[ref22] Whitfield R.; Truong N. P.; Messmer D.; Parkatzidis K.; Rolland M.; Anastasaki A. Tailoring polymer dispersity and shape of molecular weight distributions: methods and applications. Chem. Sci. 2019, 10 (38), 8724–8734. 10.1039/C9SC03546J. 33552458 PMC7844732

[ref23] Hu X.; Jazani A. M.; Oh J. K. Recent advances in development of imine-based acid-degradable polymeric nanoassemblies for intracellular drug delivery. Polymer 2021, 230, 124024 10.1016/j.polymer.2021.124024.

[ref24] Jeong J.; An S. Y.; Hu X.; Zhao Y.; Yin R.; Szczepaniak G.; Murata H.; Das S. R.; Matyjaszewski K. Biomass RNA for the Controlled Synthesis of Degradable Networks by Radical Polymerization. ACS Nano 2023, 17 (21), 21912–21922. 10.1021/acsnano.3c08244. 37851525 PMC10655241

[ref25] Matyjaszewski K.; Tsarevsky N. V. Macromolecular Engineering by Atom Transfer Radical Polymerization. J. Am. Chem. Soc. 2014, 136 (18), 6513–6533. 10.1021/ja408069v. 24758377

[ref26] Matyjaszewski K.; Tsarevsky N. V. Nanostructured functional materials prepared by atom transfer radical polymerization. Nat. Chem. 2009, 1 (4), 276–288. 10.1038/nchem.257. 21378870

[ref27] Destarac M. Controlled Radical Polymerization: Industrial Stakes, Obstacles and Achievements. Macromol. React. Eng. 2010, 4 (3–4), 165–179. 10.1002/mren.200900087.

[ref28] Matyjaszewski K.; Spanswick J. Controlled/living radical polymerization. Mater. Today 2005, 8 (3), 26–33. 10.1016/S1369-7021(05)00745-5.

[ref29] Min K.; Matyjaszewski K. Atom transfer radical polymerization in aqueous dispersed media. Central European Journal of Chemistry 2009, 7 (4), 657–674. 10.2478/s11532-009-0092-1.

[ref30] Matyjaszewski K.; Jakubowski W.; Min K.; Tang W.; Huang J.; Braunecker W. A.; Tsarevsky N. V. Diminishing catalyst concentration in atom transfer radical polymerization with reducing agents. Proc. Natl. Acad. Sci. U. S. A. 2006, 103 (42), 15309–15314. 10.1073/pnas.0602675103. 17032773 PMC1622823

[ref31] Enciso A. E.; Fu L.; Lathwal S.; Olszewski M.; Wang Z.; Das S. R.; Russell A. J.; Matyjaszewski K. Biocatalytic “Oxygen-Fueled” Atom Transfer Radical Polymerization. Angew. Chem., Int. Ed. 2018, 57 (49), 16157–16161. 10.1002/anie.201809018. 30329207

[ref32] Chapman R.; Gormley A. J.; Stenzel M. H.; Stevens M. M. Combinatorial Low-Volume Synthesis of Well-Defined Polymers by Enzyme Degassing. Angew. Chem., Int. Ed. 2016, 55 (14), 4500–4503. 10.1002/anie.201600112. 26939064

[ref33] Wang Z.; Wang Z.; Pan X.; Fu L.; Lathwal S.; Olszewski M.; Yan J.; Enciso A. E.; Wang Z.; Xia H.; Matyjaszewski K. Ultrasonication-Induced Aqueous Atom Transfer Radical Polymerization. ACS Macro Lett. 2018, 7 (3), 275–280. 10.1021/acsmacrolett.8b00027. 35632917

[ref34] McKenzie T. G.; Colombo E.; Fu Q.; Ashokkumar M.; Qiao G. G. Sono-RAFT Polymerization in Aqueous Medium. Angew. Chem., Int. Ed. 2017, 56 (40), 12302–12306. 10.1002/anie.201706771. 28834049

[ref35] Chmielarz P.; Fantin M.; Park S.; Isse A. A.; Gennaro A.; Magenau A. J. D.; Sobkowiak A.; Matyjaszewski K. Electrochemically mediated atom transfer radical polymerization (eATRP). Prog. Polym. Sci. 2017, 69, 47–78. 10.1016/j.progpolymsci.2017.02.005.

[ref36] Strover L. T.; Cantalice A.; Lam J. Y. L.; Postma A.; Hutt O. E.; Horne M. D.; Moad G. Electrochemical Behavior of Thiocarbonylthio Chain Transfer Agents for RAFT Polymerization. ACS Macro Lett. 2019, 8 (10), 1316–1322. 10.1021/acsmacrolett.9b00598. 35651172

[ref37] Magenau A. J. D.; Strandwitz N. C.; Gennaro A.; Matyjaszewski K. Electrochemically Mediated Atom Transfer Radical Polymerization. Science 2011, 332 (6025), 81–84. 10.1126/science.1202357. 21454784

[ref38] Hu X.; Szczepaniak G.; Lewandowska-Andralojc A.; Jeong J.; Li B.; Murata H.; Yin R.; Jazani A. M.; Das S. R.; Matyjaszewski K. Red-Light-Driven Atom Transfer Radical Polymerization for High-Throughput Polymer Synthesis in Open Air. J. Am. Chem. Soc. 2023, 145 (44), 24315–24327. 10.1021/jacs.3c09181. 37878520 PMC10636753

[ref39] Pan X.; Fantin M.; Yuan F.; Matyjaszewski K. Externally controlled atom transfer radical polymerization. Chem. Soc. Rev. 2018, 47 (14), 5457–5490. 10.1039/C8CS00259B. 29868657

[ref40] Lee Y.; Boyer C.; Kwon M. S. Photocontrolled RAFT polymerization: past, present, and future. Chem. Soc. Rev. 2023, 52 (9), 3035–3097. 10.1039/D1CS00069A. 37040256

[ref41] Szczepaniak G.; Jeong J.; Kapil K.; Dadashi-Silab S.; Yerneni S. S.; Ratajczyk P.; Lathwal S.; Schild D. J.; Das S. R.; Matyjaszewski K. Open-air green-light-driven ATRP enabled by dual photoredox/copper catalysis. Chem. Sci. 2022, 13 (39), 11540–11550. 10.1039/D2SC04210J. 36320395 PMC9557244

[ref42] Nothling M. D.; Fu Q.; Reyhani A.; Allison-Logan S.; Jung K.; Zhu J.; Kamigaito M.; Boyer C.; Qiao G. G. Progress and Perspectives Beyond Traditional RAFT Polymerization. Adv. Sci. 2020, 7 (20), 2001656 10.1002/advs.202001656. PMC7578854 33101866

[ref43] Kütahya C.; Schmitz C.; Strehmel V.; Yagci Y.; Strehmel B. Near-Infrared Sensitized Photoinduced Atom-Transfer Radical Polymerization (ATRP) with a Copper(II) Catalyst Concentration in the ppm Range. Angew. Chem., Int. Ed. 2018, 57 (26), 7898–7902. 10.1002/anie.201802964. 29637671

[ref44] Fors B. P.; Hawker C. J. Control of a Living Radical Polymerization of Methacrylates by Light. Angew. Chem., Int. Ed. 2012, 51 (35), 8850–8853. 10.1002/anie.201203639. 22807122

[ref45] Xu J.; Jung K.; Atme A.; Shanmugam S.; Boyer C. A Robust and Versatile Photoinduced Living Polymerization of Conjugated and Unconjugated Monomers and Its Oxygen Tolerance. J. Am. Chem. Soc. 2014, 136 (14), 5508–5519. 10.1021/ja501745g. 24689993

[ref46] Chen M.; Zhong M.; Johnson J. A. Light-Controlled Radical Polymerization: Mechanisms, Methods, and Applications. Chem. Rev. 2016, 116 (17), 10167–10211. 10.1021/acs.chemrev.5b00671. 26978484

[ref47] Jasinski F.; Zetterlund P. B.; Braun A. M.; Chemtob A. Photopolymerization in dispersed systems. Prog. Polym. Sci. 2018, 84, 47–88. 10.1016/j.progpolymsci.2018.06.006.

[ref48] Jeong J.; Hu X.; Murata H.; Szczepaniak G.; Rachwalak M.; Kietrys A.; Das S. R.; Matyjaszewski K. RNA-Polymer Hybrids via Direct and Site-Selective Acylation with the ATRP Initiator and Photoinduced Polymerization. J. Am. Chem. Soc. 2023, 145 (26), 14435–14445. 10.1021/jacs.3c03757. 37357749 PMC10326879

[ref49] Ciftci M.; Tasdelen M. A.; Li W.; Matyjaszewski K.; Yagci Y. Photoinitiated ATRP in Inverse Microemulsion. Macromolecules 2013, 46 (24), 9537–9543. 10.1021/ma402058a.

[ref50] Jung K.; Xu J.; Zetterlund P. B.; Boyer C. Visible-Light-Regulated Controlled/Living Radical Polymerization in Miniemulsion. ACS Macro Lett. 2015, 4 (10), 1139–1143. 10.1021/acsmacrolett.5b00576. 35614795

[ref51] Jung K.; Boyer C.; Zetterlund P. B. RAFT iniferter polymerization in miniemulsion using visible light. Polym. Chem. 2017, 8 (27), 3965–3970. 10.1039/C7PY00939A.

[ref52] Davidson C. L. G. I. V.; Lott M. E.; Trachsel L.; Wong A. J.; Olson R. A.; Pedro D. I.; Sawyer W. G.; Sumerlin B. S. Inverse Miniemulsion Enables the Continuous-Flow Synthesis of Controlled Ultra-High Molecular Weight Polymers. ACS Macro Lett. 2023, 12 (9), 1224–1230. 10.1021/acsmacrolett.3c00431. 37624643

[ref53] Wang Y.; Dadashi-Silab S.; Matyjaszewski K. Photoinduced Miniemulsion Atom Transfer Radical Polymerization. ACS Macro Lett. 2018, 7 (6), 720–725. 10.1021/acsmacrolett.8b00371. 35632954

[ref54] Qiao X.; Qiao L.; Zhou M.; Zhang X.; Shi G.; He Y.; Bourgeat-Lami E.; Pang X. Carbon Quantum Dot-Catalyzed, Highly Efficient Miniemulsion Atom Transfer Radical Polymerization Induced by Visible Light. ACS Macro Lett. 2022, 11 (11), 1298–1305. 10.1021/acsmacrolett.2c00542. 36326145

[ref55] Olson R. A.; Lott M. E.; Garrison J. B.; Davidson C. L. G. I. V.; Trachsel L.; Pedro D. I.; Sawyer W. G.; Sumerlin B. S. Inverse Miniemulsion Photoiniferter Polymerization for the Synthesis of Ultrahigh Molecular Weight Polymers. Macromolecules 2022, 55 (19), 8451–8460. 10.1021/acs.macromol.2c01239.

[ref56] Oh J. K.; Tang C.; Gao H.; Tsarevsky N. V.; Matyjaszewski K. Inverse Miniemulsion ATRP: A New Method for Synthesis and Functionalization of Well-Defined Water-Soluble/Cross-Linked Polymeric Particles. J. Am. Chem. Soc. 2006, 128 (16), 5578–5584. 10.1021/ja060586a. 16620132

[ref57] Yin R.; Zhao Y.; Jeong J.; Tarnsangpradit J.; Liu T.; An S. Y.; Zhai Y.; Hu X.; Bockstaller M. R.; Matyjaszewski K. Composition-Orientation Induced Mechanical Synergy in Nanoparticle Brushes with Grafted Gradient Copolymers. Macromolecules 2023, 56 (23), 9626–9635. 10.1021/acs.macromol.3c01799. 38105929 PMC10720466

[ref58] Wang Y.; Dadashi-Silab S.; Lorandi F.; Matyjaszewski K. Photoinduced atom transfer radical polymerization in ab initio emulsion. Polymer 2019, 165, 163–167. 10.1016/j.polymer.2019.01.034.

[ref59] Fan W.; Tosaka M.; Yamago S.; Cunningham M. F. Living Ab Initio Emulsion Polymerization of Methyl Methacrylate in Water Using a Water-Soluble Organotellurium Chain Transfer Agent under Thermal and Photochemical Conditions. Angew. Chem., Int. Ed. 2018, 57 (4), 962–966. 10.1002/anie.201710754. 29124836

[ref60] Truong N. P.; Quinn J. F.; Anastasaki A.; Rolland M.; Vu M. N.; Haddleton D. M.; Whittaker M. R.; Davis T. P. Surfactant-free RAFT emulsion polymerization using a novel biocompatible thermoresponsive polymer. Polym. Chem. 2017, 8 (8), 1353–1363. 10.1039/C6PY02158A.

[ref61] Lorandi F.; Wang Y.; Fantin M.; Matyjaszewski K. Ab Initio Emulsion Atom-Transfer Radical Polymerization. Angew. Chem., Int. Ed. 2018, 57 (27), 8270–8274. 10.1002/anie.201804647. 29845718

[ref62] Shim S. E.; Jung H.; Lee H.; Biswas J.; Choe S. Living radical dispersion photopolymerization of styrene by a reversible addition–fragmentation chain transfer (RAFT) agent. Polymer 2003, 44 (19), 5563–5572. 10.1016/S0032-3861(03)00632-3.

[ref63] Ki B.; Yu Y. C.; Jeon H. J.; Yu W.-R.; Ryu H. W.; Youk J. H. Dispersion polymerization of styrene using poly(4-vinylpyridine) macro-RAFT agent under UV radiation. Fibers Polym. 2012, 13 (1), 135–138. 10.1007/s12221-012-0135-7.

[ref64] Tan J.; Rao X.; Wu X.; Deng H.; Yang J.; Zeng Z. Photoinitiated RAFT Dispersion Polymerization: A Straightforward Approach toward Highly Monodisperse Functional Microspheres. Macromolecules 2012, 45 (21), 8790–8795. 10.1021/ma301799r.

[ref65] Wang G.; Xi M.; Bai L.; Liang Y.; Yang L.; Wang W.; Chen H.; Yang H. Pickering emulsion of metal-free photoinduced electron transfer-ATRP stabilized by cellulose nanocrystals. Cellulose 2019, 26 (10), 5947–5957. 10.1007/s10570-019-02528-4.

[ref66] Cuneo T.; Wang X.; Shi Y.; Gao H. Synthesis of Hyperbranched Polymers via Metal-Free ATRP in Solution and Microemulsion. Macromol. Chem. Phys. 2020, 221 (6), 2000008 10.1002/macp.202000008.

[ref67] Yeow J.; Chapman R.; Gormley A. J.; Boyer C. Up in the air: oxygen tolerance in controlled/living radical polymerisation. Chem. Soc. Rev. 2018, 47 (12), 4357–4387. 10.1039/C7CS00587C. 29718038 PMC9857479

[ref68] Szczepaniak G.; Fu L.; Jafari H.; Kapil K.; Matyjaszewski K. Making ATRP More Practical: Oxygen Tolerance. Acc. Chem. Res. 2021, 54 (7), 1779–1790. 10.1021/acs.accounts.1c00032. 33751886

[ref69] Hou H.; Tang D.; Zhang L.; Zhao D.; Xiao H.; Li B. NIR light triggered intracellular polymerization via nanoparticles containing acrylates prodrugs and azo-polymers for inhibiting cisplatin efflux for combined chemotherapy and immunotherapy. Nano Today 2023, 50, 101858 10.1016/j.nantod.2023.101858.

[ref70] Cabanero D. C.; Kariofillis S. K.; Johns A. C.; Kim J.; Ni J.; Park S.; Parker D. L. Jr.; Ramil C. P.; Roy X.; Shah N. H.; Rovis T. Photocatalytic Activation of Aryl(trifluoromethyl) Diazos to Carbenes for High-Resolution Protein Labeling with Red Light. J. Am. Chem. Soc. 2024, 146 (2), 1337–1345. 10.1021/jacs.3c09545. 38165744

[ref71] Tay N. E. S.; Ryu K. A.; Weber J. L.; Olow A. K.; Cabanero D. C.; Reichman D. R.; Oslund R. C.; Fadeyi O. O.; Rovis T. Targeted activation in localized protein environments via deep red photoredox catalysis. Nat. Chem. 2023, 15 (1), 101–109. 10.1038/s41557-022-01057-1. 36216892 PMC9840673

[ref72] Wu Z.; Boyer C. Near-Infrared Light-Induced Reversible Deactivation Radical Polymerization: Expanding Frontiers in Photopolymerization. Adv. Sci. 2023, 10, 2304942 10.1002/advs.202304942. PMC10667859 37750445

[ref73] Wu Z.; Fang W.; Wu C.; Corrigan N.; Zhang T.; Xu S.; Boyer C. An aqueous photo-controlled polymerization under NIR wavelengths: synthesis of polymeric nanoparticles through thick barriers. Chem. Sci. 2022, 13 (39), 11519–11532. 10.1039/D2SC03952D. 36320386 PMC9555728

[ref74] Zhang W.; He J.; Lv C.; Wang Q.; Pang X.; Matyjaszewski K.; Pan X. Atom Transfer Radical Polymerization Driven by Near-Infrared Light with Recyclable Upconversion Nanoparticles. Macromolecules 2020, 53 (12), 4678–4684. 10.1021/acs.macromol.0c00850.

[ref75] Qiao X.; Hao Q.; Chen M.; Shi G.; He Y.; Pang X. Simple Full-Spectrum Heterogeneous Photocatalyst for Photo-induced Atom Transfer Radical Polymerization (ATRP) under UV/vis/NIR and its Application for the Preparation of Dual Mode Curing Injectable Photoluminescence Hydrogel. ACS Appl. Mater. Interfaces 2022, 14 (18), 21555–21563. 10.1021/acsami.2c04065. 35500109

[ref76] Patel R. I.; Sharma A.; Sharma S.; Sharma A. Visible light-mediated applications of methylene blue in organic synthesis. Org. Chem. Front. 2021, 8 (7), 1694–1718. 10.1039/D0QO01182G.

[ref77] Aguirre-Soto A.; Lim C.-H.; Hwang A. T.; Musgrave C. B.; Stansbury J. W. Visible-Light Organic Photocatalysis for Latent Radical-Initiated Polymerization via 2e–/1H+ Transfers: Initiation with Parallels to Photosynthesis. J. Am. Chem. Soc. 2014, 136 (20), 7418–7427. 10.1021/ja502441d. 24786755 PMC4046762

[ref78] Mohamed Irshadeen I.; Truong V. X.; Frisch H.; Barner-Kowollik C. Red light induced folding of single polymer chains. Chem. Commun. 2022, 58 (93), 12975–12978. 10.1039/D2CC05415A. 36326031

[ref79] Fantin M.; Chmielarz P.; Wang Y.; Lorandi F.; Isse A. A.; Gennaro A.; Matyjaszewski K. Harnessing the Interaction between Surfactant and Hydrophilic Catalyst To Control eATRP in Miniemulsion. Macromolecules 2017, 50 (9), 3726–3732. 10.1021/acs.macromol.7b00530. 29977099 PMC6029256

[ref80] Simakova A.; Averick S. E.; Konkolewicz D.; Matyjaszewski K. Aqueous ARGET ATRP. Macromolecules 2012, 45 (16), 6371–6379. 10.1021/ma301303b.

[ref81] Teo V. L.; Davis B. J.; Tsarevsky N. V.; Zetterlund P. B. Successful Miniemulsion ATRP Using an Anionic Surfactant: Minimization of Deactivator Loss by Addition of a Halide Salt. Macromolecules 2014, 47 (18), 6230–6237. 10.1021/ma501379q.

[ref82] Jazani A. M.; Schild D. J.; Sobieski J.; Hu X.; Matyjaszewski K. Visible Light-ATRP Driven by Tris(2 pyridylmethyl)amine (TPMA) Impurities in the Open Air. Macromol. Rapid Commun. 2022, 44 (16), 2200855 10.1002/marc.202200855. 36471106

[ref83] Spatorico A. L.; Coulter B. Molecular weight determinations by gel-permeation chromatography and viscometry. J. Polym. Sci., Polym. Phys. Ed. 1973, 11 (6), 1139–1150. 10.1002/pol.1973.180110608.

[ref84] Gruendling T.; Junkers T.; Guilhaus M.; Barner-Kowollik C. Mark–Houwink Parameters for the Universal Calibration of Acrylate, Methacrylate and Vinyl Acetate Polymers Determined by Online Size-Exclusion Chromatography—Mass Spectrometry. Macromol. Chem. Phys. 2010, 211 (5), 520–528. 10.1002/macp.200900323.

[ref85] Zetterlund P. B. Controlled/living radical polymerization in nanoreactors: compartmentalization effects. Polym. Chem. 2011, 2 (3), 534–549. 10.1039/C0PY00247J.

[ref86] Krishnan S.; Klein A.; El-Aasser M. S.; Sudol E. D. Effect of Surfactant Concentration on Particle Nucleation in Emulsion Polymerization of n-Butyl Methacrylate. Macromolecules 2003, 36 (9), 3152–3159. 10.1021/ma021120p.

[ref87] Pellegrin Y.; Odobel F. Sacrificial electron donor reagents for solar fuel production. Comptes Rendus Chimie 2017, 20 (3), 283–295. 10.1016/j.crci.2015.11.026.

[ref88] Melgoza D.; Hernández-Ramírez A.; Peralta-Hernández J. Comparative efficiencies of the decolourisation of Methylene Blue using Fenton’s and photo-Fenton’s reactions. Photochem. Photobiol. Sci. 2009, 8, 596–599. 10.1039/b817287k. 19424530

[ref89] De Bon F.; Barbosa A. B.; Fonseca R. G.; Fantin M.; Serra A. C.; Coelho J. F. J. Large volume and oxygen tolerant photoinduced aqueous atom transfer radical polymerization. Chem. Eng. J. 2023, 451, 138777 10.1016/j.cej.2022.138777.

