# Supplementary material for: Robust Miniemulsion PhotoATRP Driven by Red and Near-Infrared Light
Source: J Am Chem Soc. 2024 May 1;146(19):13417–26. doi: 10.1021/jacs.4c02553 (PMC11099965; doi:10.1021/jacs.4c02553)
Supplement: Supplementary file 1 — ja4c02553_si_001.pdf [file ja4c02553_si_001.pdf]

Supporting Information

Robust Miniemulsion PhotoATRP Driven by Red and Near-Infrared Light

Xiaolei Hu, Rongguan Yin, Jaepil Jeong,* and Krzysztof Matyjaszewski*

Department of Chemistry, Carnegie Mellon University, Pittsburgh, Pennsylvania 15213, USA

Table of Contents

Materials and Instruments ... *3*

Materials ... 3

Instruments ... 3

Experimental Procedures ... *4*

General procedure for miniemulsion photoATRP of BMA under NIR light irradiation ... 4

Kinetic study under varying amounts of $CuBr_2$/TPMA complex in Table 1 ... 9

Miniemulsion photoATRP using different light wavelengths ... 11

Homogeneous photoATRP under different light wavelengths ... 13

Chain extension ... 15

Synthesis of pBMA with varying DP_T ... 16

Temporal control under NIR light ... 17

Temporal control under red light ... 18

Miniemulsion photoATRP in reactors with different diameters under different light wavelengths ... 19

Miniemulsion photoATRP passing through an A4 paper under red and NIR light ... 22

Miniemulsion photoATRP in a large scale (250 mL) ... 23

References ... *24*

Materials and Instruments

Materials

Unless otherwise noted, all chemicals were purchased from commercial sources and used as received. Methylene blue (MB^+, 99%), copper(II) bromide ($CuBr_2$, 99.99%), ethyl α-bromophenylacetate (EBPA, ≥99%), 2-hydroxyethyl α-bromoisobutyrate (HO-EBiB, 95%), n-butyl methacrylate (BMA, 99%), n-butyl acrylate (BA, ≥99%), oligo(ethylene glycol) methyl ether methacrylate (average M_n = 500, $OEOMA_{500}$), sodium bromide (NaBr, ≥99%), triethanolamine (TEOA, ≥99%), sodium dodecyl sulfate (SDS, 99%), hexadecane (HD, 99%), were purchased from *Sigma-Aldrich*. All monomers were passed through a column of basic alumina to remove the inhibitor before use. Tris(2-pyridylmethyl)amine (TPMA, 99%) was purchased from *AmBeed*. 10X phosphate-buffered saline (10X PBS) was purchased from *Thermo Fisher Scientific*. Water (HPLC grade), dimethylformamide (DMF, HPLC grade), tetrahydrofuran (THF, HPLC grade), and dimethyl sulfoxide (DMSO, HPLC grade) were purchased from *Fisher Chemical*. D_2O was purchased from *Cambridge Isotope Laboratories, Inc.*

Instruments

Sonicator

Ultrasound treatment to prepare miniemulsion samples was carried out using Autotune Series High Intensity Ultrasonic Processor, 1500-Watt Model.

Photoreactor for miniemulsion photoATRP

Polymerization was conducted in a EvoluChem™ PhotoRedOx Box purchased from *Hepatochem* with varying LEDs. LEDs with UV light (390 nm, 25 mW cm^{-2}), green light (520 nm, 25 mW cm^{-2}), and red light (640 nm, 25 mW cm^{-2}) were purchased from *Kessil* except for NIR lights (740 nm or 808 nm, 20 mW cm^{-2}) from *Hepatochem.*

Gravimetric analysis for the measurement of monomer conversion

Monomer conversion during miniemulsion photoATRP was determined by gravimetric analysis after evaporating the samples (100 μL) at 110 °C for at least 1 h.[1]

Size exclusion chromatography (SEC)

SEC measurements of pBMA were conducted using *PSS* columns (Styrogel 10^2, 10^3, 10^4, 10^5 Å) with THF as the eluent at 35 °C and the flow rate of 1 mL/min. Linear polystyrene standards were used for SEC calibration. Absolute molecular weight ($M_{n,abs}$) was determined by Mark-Houwink calibration with K and a values of PS and pBMA from the literature.[2, 3] SEC measurements of $pOEOMA_{500}$ were performed using *PSS* columns (Styrogel 10^5, 10^3, 10^2 Å) with DMF containing LiBr (0.05 M) as the eluent at 50 °C and the flow rate of 1 mL/min.

Dynamic light scattering (DLS)

Particle sizes were determined by using a Zetasizer Nano from *Malvern Instruments, Ltd*.

^{1}H Nuclear magnetic resonance (^{1}H NMR)

^{1}H NMR spectra were recorded on *Bruker Avance III* 500 MHz spectrometer with D_2O used as the solvent.

Experimental Procedures

General procedure for miniemulsion photoATRP of BMA under NIR light irradiation

First, stock solutions of EBPA/BMA (31.5 mM of EBPA in BMA), $CuBr_2$/TPMA complex (1:1 molar ratio, 50 mM in H_2O), MB^+ (1.88 mM in H_2O), TEOA (100 mM in H_2O), NaBr (2 M in H_2O), and SDS (300 mM in H_2O) were prepared. A typical ATRP “cocktail” mixture was then prepared as follows. EBPA/BMA stock (1007 µL), HD (125 µL), MB^+ stock (420 µL), $CuBr_2$/TPMA stock (63 µL), TEOA stock (189 µL), NaBr stock (250 µL), SDS stock (950 µL), and H_2O (2 mL) were then mixed (total volume = 5 mL). The final concentrations were BMA (1.26 M), MB^+ (15.7 µM), $CuBr_2$/TPMA (0.63 mM), EBPA (6.3 mM), and TEOA (3.77 mM). The ATRP “cocktail” was homogenized by a probe sonication (amplitude = 25%, application and rest time of 1 s each) for 10 min under an ice-water bath. The one-dram vial (diameter = 15 mm) with ATRP “cocktail” miniemulsion mixture equipped with a magnetic stirring was mounted on the EvoluChem™ PhotoRedOx Box. The polymerization mixtures were irradiated under NIR light LEDs (740 nm, 20 mW cm^{-2}) for 60 min under stirring (500 rpm) in open air. Samples were withdrawn periodically during the polymerization for gravimetric analysis and SEC characterization.

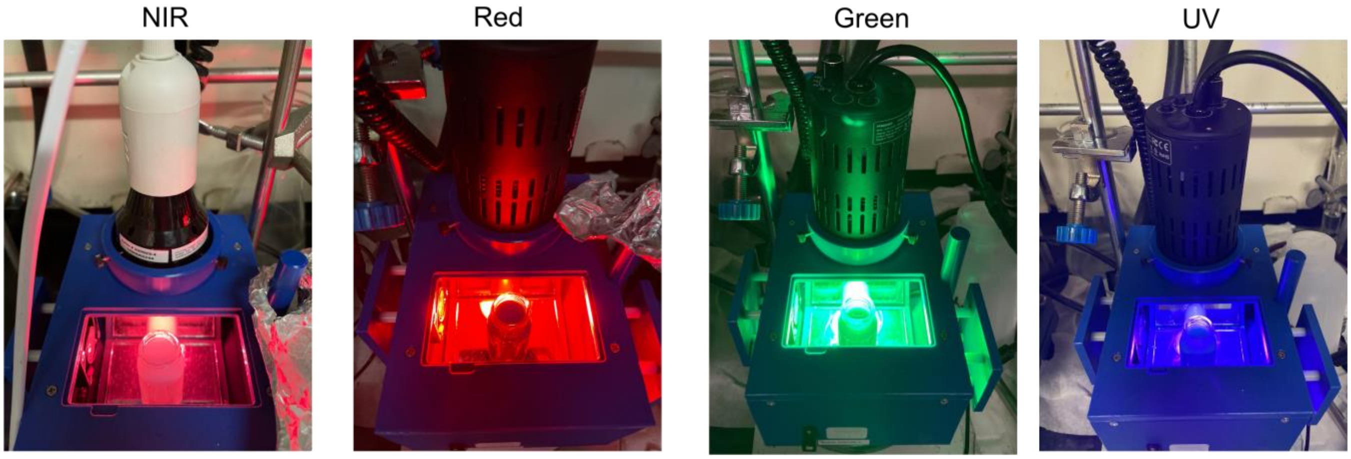

Figure S1. Digital camera images demonstrating the set up for the miniemulsion photoATRP under different light sources: NIR LED (λ_{max} = 740 nm, 20 mW cm^{-2}), red LED (λ_{max} = 640 nm, 25 mW cm^{-2}), green LED (λ_{max} = 520 nm, 25 mW cm^{-2}), and UV LED (λ_{max} = 390 nm, 25 mW cm^{-2}), respectively.

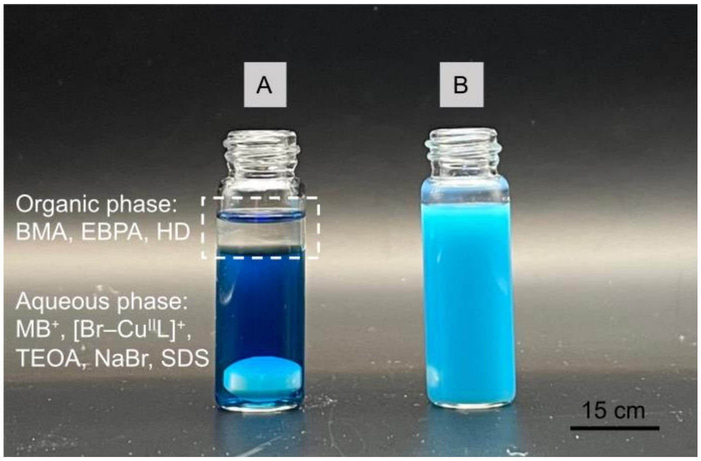

Figure S2. Digital camera image of miniemulsion photoATRP "cocktail" (A) before; and (B) after sonication in a one-dram vial (diameter = 15 mm), respectively.

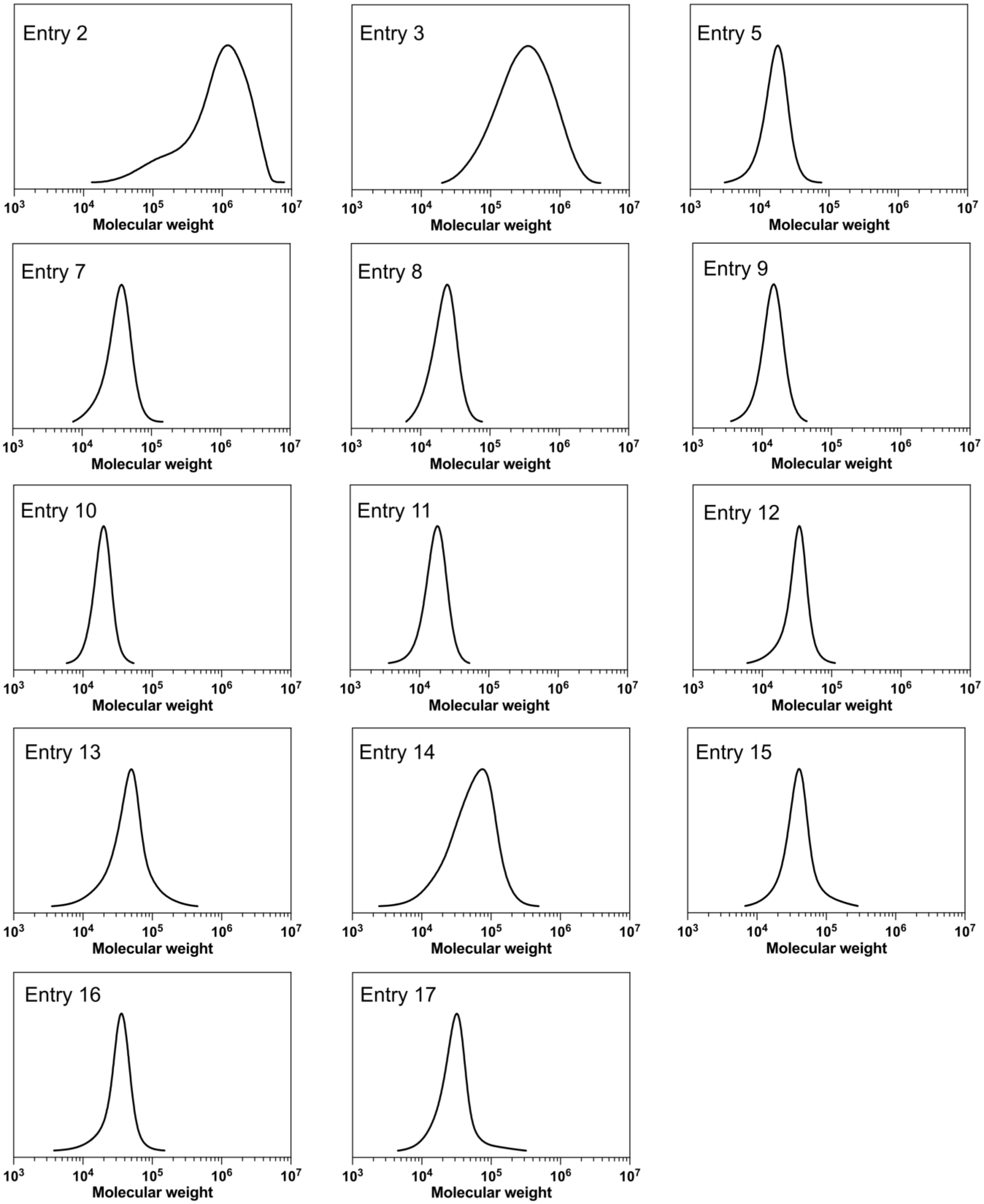

Figure S3. SEC traces for the miniemulsion photoATRP results shown in Table 1.

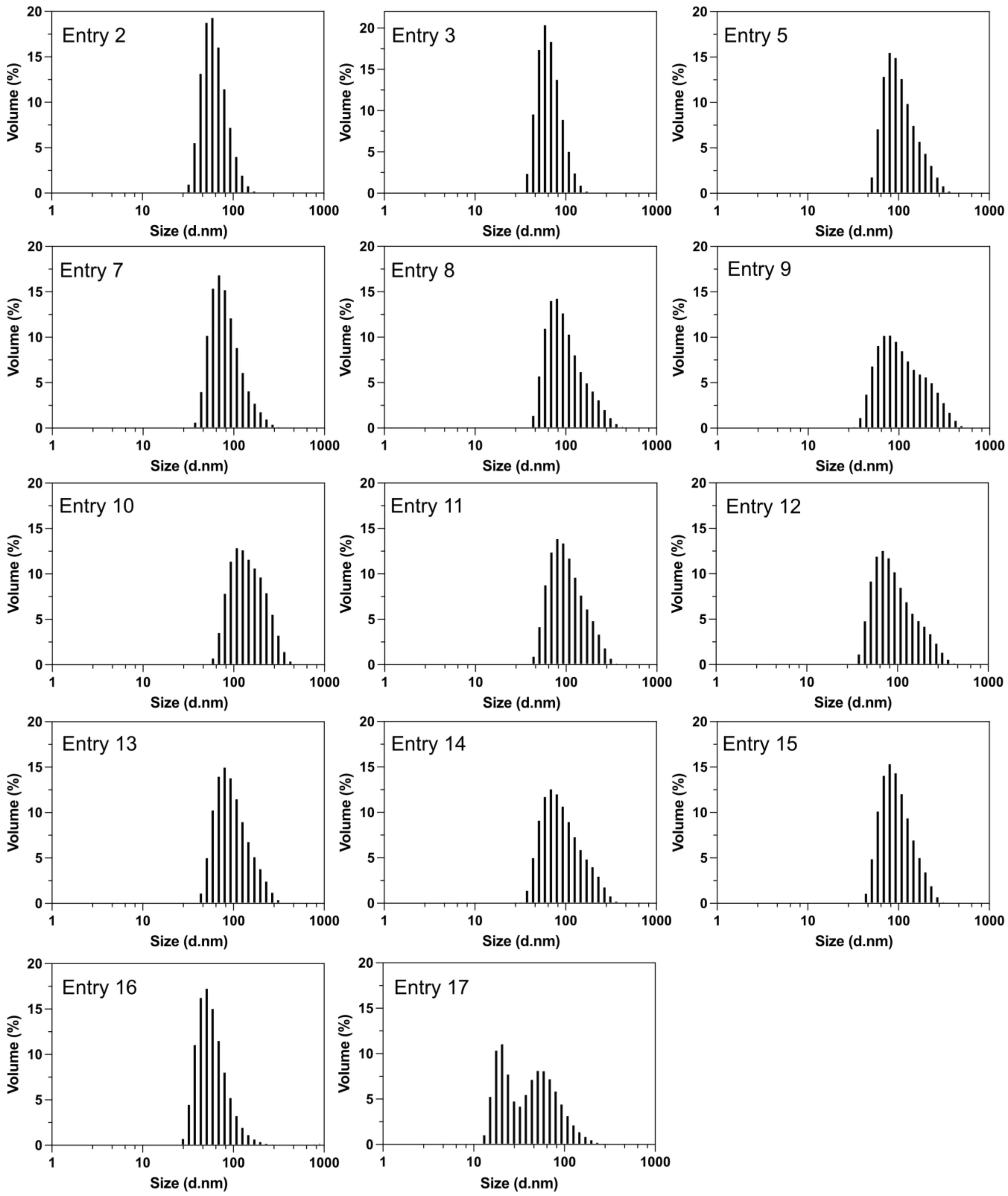

Figure S4. DLS results for the miniemulsion photoATRP results shown in Table 1.

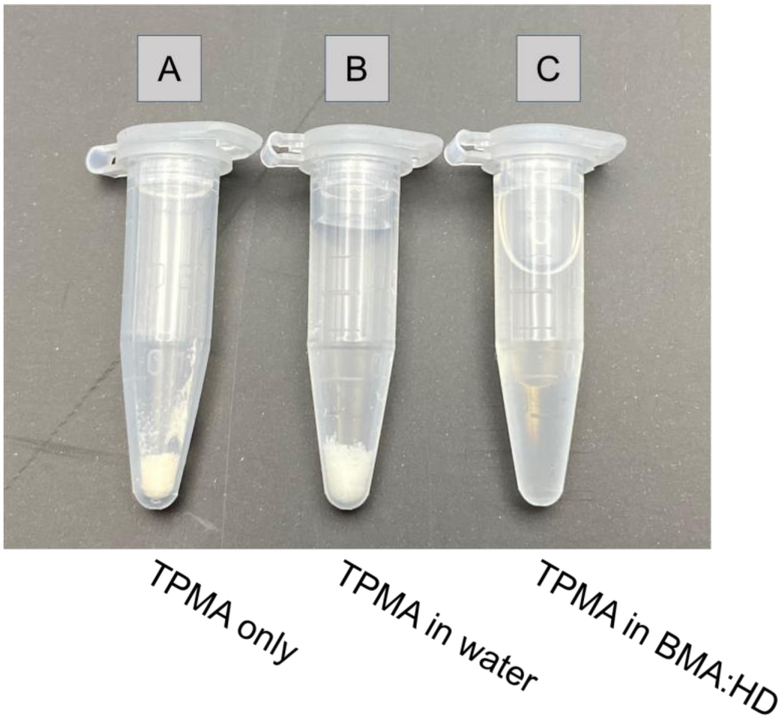

Figure S5. Digital camera image of the TPMA solubility comparison. (A) 5.5 mg of TPMA; (B) 5.5 mg of TPMA in 500 µL of water; and (C) 5.5 mg of TPMA in the 500 µL of organic phase (BMA:HD = 1:0.125 , v/v).

Kinetic study under varying amounts of $CuBr_2$/TPMA complex in Table 1

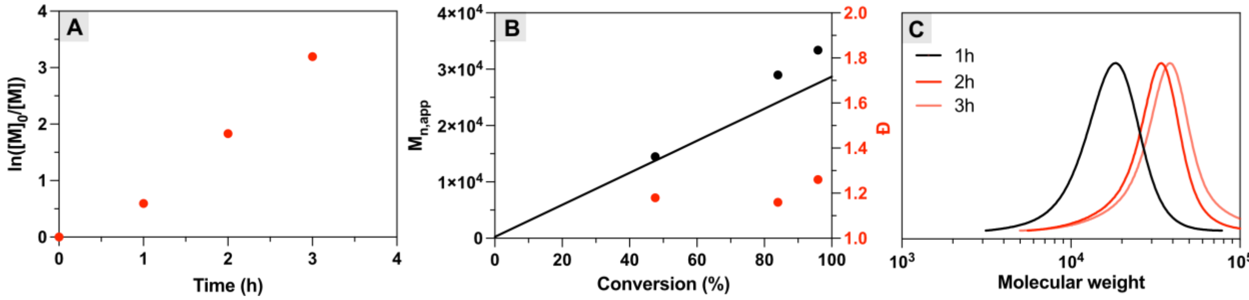

Figure S6. Kinetic study for the reaction in Entry 5, Table 1. (A) first-order kinetic plot; (B) evolution of molecular weight and molecular weight distribution with monomer conversion; and (C) SEC traces evolution with time. Reaction conditions: [BMA]/[EBPA]/[MB^+]/[$CuBr_2$/TPMA]/[TEOA] = 200/1/0.025/0.1/0.6, [M] = 20 vol% to total, [HD] =10.8 wt % to BMA, [SDS] = 4.6 wt% relative to BMA, [NaBr] = 0.1 M, irradiated under red LED (640 nm, 25 mW cm^{-2}) in a one-dram vial (diameter = 15 mm) in open air under stirring (500 rpm).

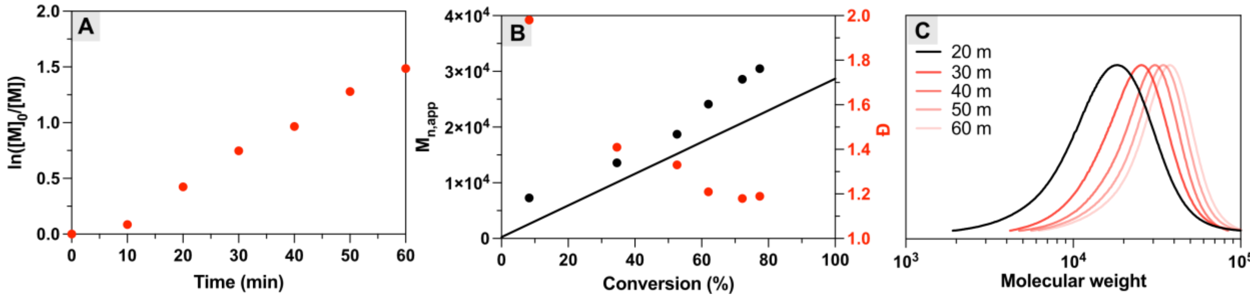

Figure S7. Kinetic study of the reaction in Entry 7, Table 1. (A) first-order kinetic plot; (B) evolution of molecular weight and molecular weight distribution with monomer conversion; and (C) SEC traces evolution over time. Reaction conditions: [BMA]/[EBPA]/[MB^+]/[$CuBr_2$/TPMA]/[TEOA] = 200/1/0.025/0.025/0.6, [M] = 20 vol% to total, [HD] =10.8 wt % to BMA, [SDS] = 4.6 wt% relative to BMA, [NaBr] = 0.1 M, irradiated under red LED (640 nm, 25 mW cm^{-2}) in a one-dram vial (diameter = 15 mm) in open air under stirring (500 rpm).

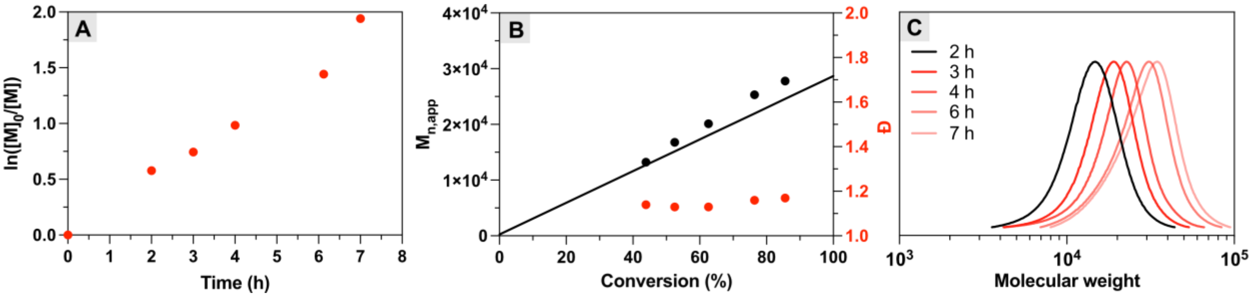

Figure S8. Kinetic study of the reaction in Entry 9, Table 1. (A) first-order kinetic plot; (B) evolution of molecular weight and molecular weight distribution with monomer conversion; and (C) SEC traces evolution over time. Reaction conditions: [BMA]/[EBPA]/[MB^+]/[$CuBr_2$/TPMA]/[TEOA] = 200/1/0.025/0.2/0.6, [M] = 20 vol% to total, [HD] =10.8 wt % to BMA, [SDS] = 4.6 wt% relative to BMA, [NaBr] = 0.1 M, irradiated under red LED (640 nm, 25 mW cm^{-2}) in a one-dram vial (diameter = 15 mm) in open air under stirring (500 rpm).

Miniemulsion photoATRP using different light wavelengths

The miniemulsion ATRP "cocktail" mixture (5 mL) was prepared based according to the general procedure for MB^+/Cu-catalyzed photoATRP at final concentrations of BMA (1.26 M), MB^+ (15.7 µM), $CuBr_2$/TPMA (0.63 mM), EBPA (6.3 mM), and TEOA (3.77 mM). The polymerization mixture was transferred to a one-dram vial (diameter = 15 mm) equipped with a magnetic stirring bar. The vial was mounted on the EvoluChem™ PhotoRedOx Box and irradiated under a different light wavelengths (UV light: 390 nm, 25 mW cm^{-2}, green light: 520 nm, 25 mW cm^{-2}, red light: 640 nm, 25 mW cm^{-2}, and NIR light: 740 nm, 20 mW cm^{-2}) under stirring (500 rpm) in open air. Samples were withdrawn periodically for gravimetric analysis and SEC measurement.

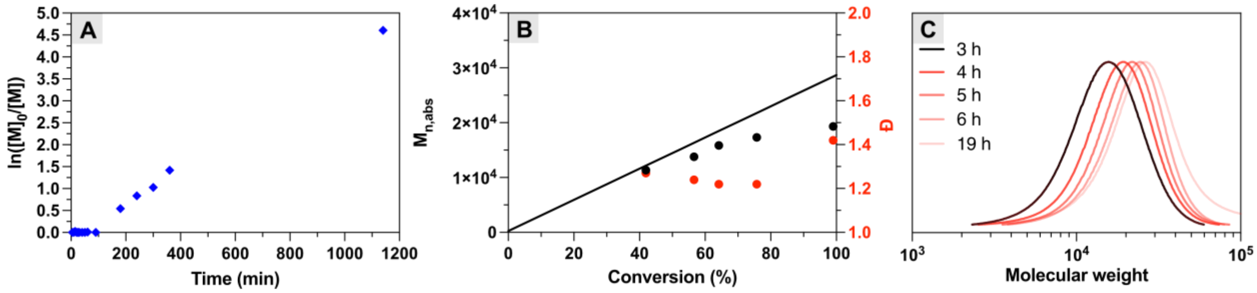

Figure S9. PhotoATRP in miniemulsion under UV light. (A) first-order kinetic plot; (B) evolution of molecular weight and molecular weight distribution with monomer conversion; and (C) SEC traces evolution with time. Reaction conditions: [BMA]/[EBPA]/[MB^+]/[$CuBr_2$/TPMA]/[TEOA] = 200/1/0.025/0.1/0.6, [M] = 20 vol% to total, [HD] =10.8 wt % to BMA, [SDS] = 9.2 wt% relative to BMA, [NaBr] = 0.1 M, irradiated under UV light (390 nm, 25 mW cm^{-2}) in a one-dram vial (diameter = 15 mm) in open air under stirring (500 rpm).

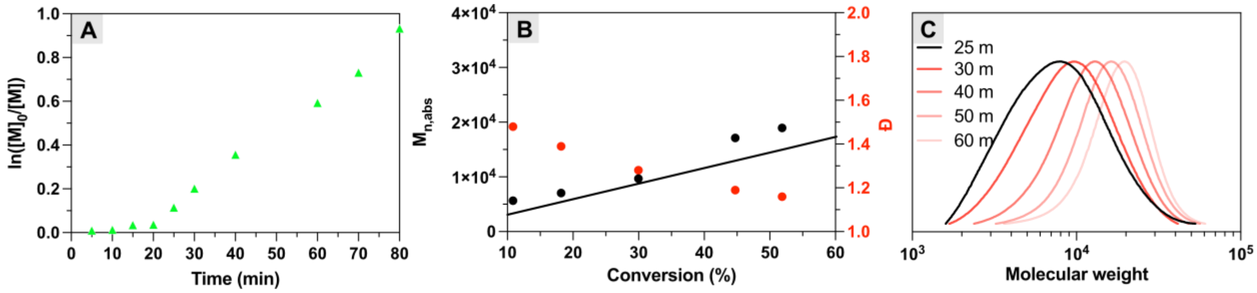

Figure S10. PhotoATRP in miniemulsion under green light. (A) first-order kinetic plot; (B) evolution of molecular weight and molecular weight distribution with monomer conversion; and (C) SEC traces evolution over time. Reaction conditions: [BMA]/[EBPA]/[MB^+]/[$CuBr_2$/TPMA]/[TEOA] = 200/1/0.025/0.1/0.6, [M] = 20 vol% to total, [HD] =10.8 wt % to BMA, [SDS] = 9.2 wt% relative to BMA, [NaBr] = 0.1 M, irradiated under green light (520 nm, 25 mW cm^{-2}) in a one-dram vial (diameter = 15 mm) in open air under stirring (500 rpm).

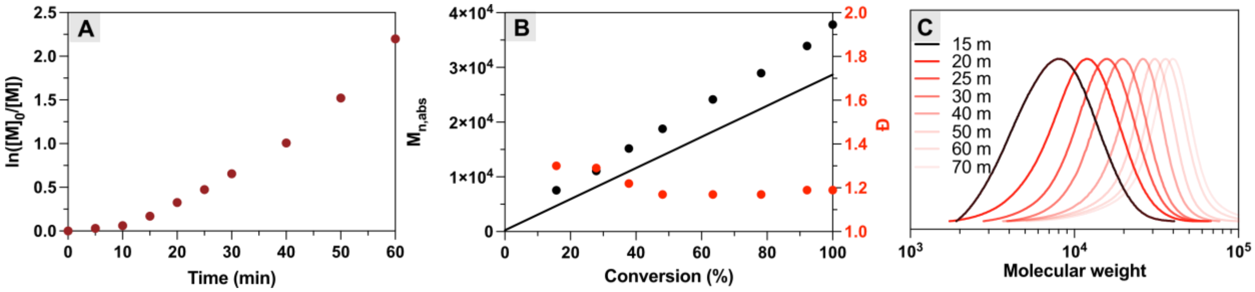

Figure S11. PhotoATRP in miniemulsion under red light. (A) first-order kinetic plot; (B) evolution of molecular weight and molecular weight distribution with monomer conversion; and (C) SEC traces evolution over time. Reaction conditions: [BMA]/[EBPA]/[MB$^+$]/[$CuBr_2$/TPMA]/[TEOA] = 200/1/0.025/0.1/0.6, [M] = 20 vol% to total, [HD] =10.8 wt % to BMA, [SDS] = 9.2 wt% relative to BMA, [NaBr] = 0.1 M, irradiated under red light (640 nm, 25 mW cm^{-2}) in a one-dram vial (diameter = 15 mm) in open air under stirring (500 rpm).

Homogeneous photoATRP under different light wavelengths

The homogeneous aqueous polymerization of $OEOMA_{500}$ was performed following the previously reported procedure.[4] First, stock solutions of $OEOMA_{500}$ (600 mM in H_2O), MB^+ (1.88 mM in H_2O), HO-EBiB (75 mM in H_20), $CuBr_2$ (11.25 mM in H_2O), and TPMA (67.49 mM in DMSO) were prepared. A typical ATRP "cocktail" solution (5 mL) was then prepared as follows. $OEOMA_{500}$ stock (2.5 mL), MB^+ stock (0.1 mL), $CuBr_2$ stock (0.2 mL), TPMA stock (0.1 mL), HO-EBiB stock (0.1 mL), DMSO (0.4 mL), H_2O (1.1 µL) and 10X PBS solution (0.5 mL) were then mixed. The final concentrations were $OEOMA_{500}$ (300 mM), MB^+ (37.5 µM), $CuBr_2$ (0.45 mM), TPMA (1.35 mM), HO-EBiB (1.5 mM), DMSO (10% *v*/*v*), and 1X PBS. The ATRP "cocktail" was then transferred to a one-dram vial (diameter = 15 mm) equipped with a magnetic stirring bar. The vial was mounted on the EvoluChem™ PhotoRedOx Box and irradiated under a different light (UV light: 390 nm, 25 mW cm^{-2}, green light: 520 nm, 25 mW cm^{-2}, red light: 640 nm, 25 mW cm^{-2}, and NIR light: 740 nm, 20 mW cm^{-2}) under stirring (500 rpm) in open air. At different time intervals, samples were withdrawn for ^1H NMR analysis and SEC measurement.

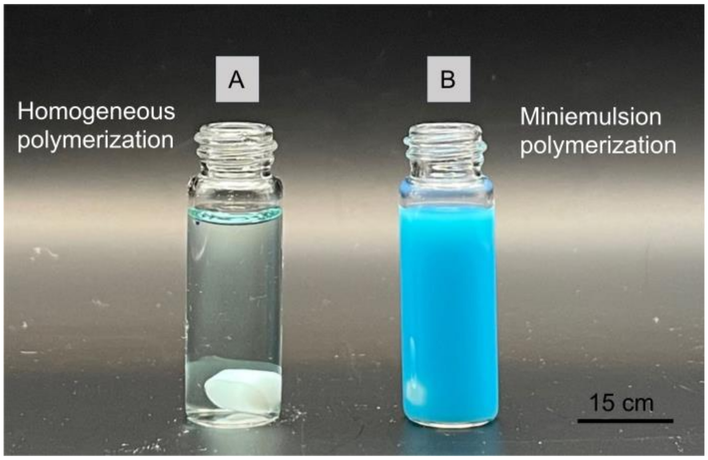

Figure S12. Digital camera image of photoATRP reaction mixtures under (A) homogeneous aqueous solution and (B) miniemulsion condition in a one-dram vial (diameter = 15 mm), respectively.

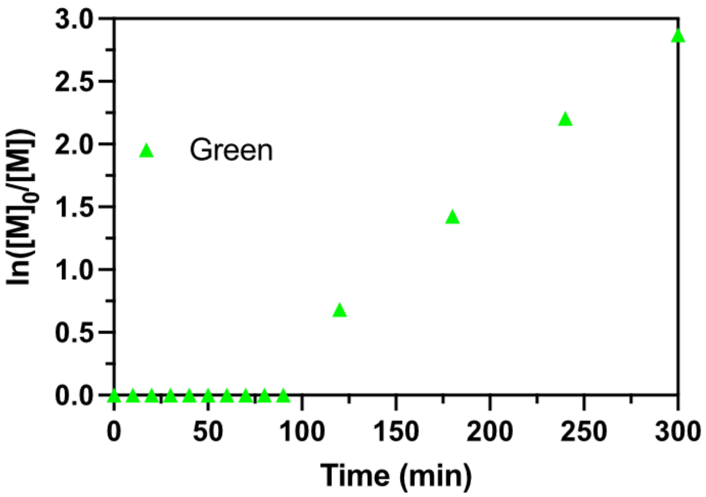

Figure S13. First-order kinetic plot for the photoATRP of $OEOMA_{500}$ in homogeneous aqueous phase under the irradiation of green light.

Chain extension

For the synthesis of pBMA-*b*-pBA block copolymer, pBMA with DP_T = 50 was first synthesized under the condition of [BMA]/[EPBA]/[MB^+]/[$CuBr_2$/TPMA]/[TEOA] = 50/1/0.025/0.1/0.6 and irradiation of NIR LEDs (740 nm, 20 mW cm^{-2}) for 40 min under stirring (500 rpm) in open air. Subsequently, the crude macroinitiator sample pBMA taken from the post-polymerization solution was used directly after polymerization to prepare the ATRP "cocktail" for chain extension with BA at DP_T = 280 under [BA]/[pBMA]/[MB^+]/[$CuBr_2$/TPMA]/[TEOA] = 280/1/0.025/0.1/0.6. The polymerization mixture was irradiated under NIR LEDs (740 nm, 20 mW cm^{-2}) for 60 min under stirring (500 rpm) in open air. Finally, the sample was withdrawn for gravimetric analysis and SEC measurement.

For the synthesis of pBMA-*b*-pBMA block copolymer, pBMA with DP_T = 50 was first synthesized under the condition of [BMA]/[EPBA]/[MB^+]/[$CuBr_2$/TPMA]/[TEOA] = 50/1/0.025/0.1/0.6 and irradiation of NIR LEDs (740 nm, 20 mW cm^{-2}) for 40 min under stirring (500 rpm) in open air. Subsequently, the crude macroinitiator sample pBMA taken from the post-polymerization solution was used directly after polymerization to prepare the ATRP "cocktail" for chain extension with BMA at DP_T = 250 under the condition of [BMA]/[pBMA]/[MB^+]/[$CuBr_2$/TPMA]/[TEOA] = 250/1/0.025/0.1/0.6. The polymerization mixture was irradiated under NIR LEDs (740 nm, 20 mW cm^{-2}) for 60 min under stirring (500 rpm) in open air. Finally, the sample was withdrawn for gravimetric analysis and SEC measurement.

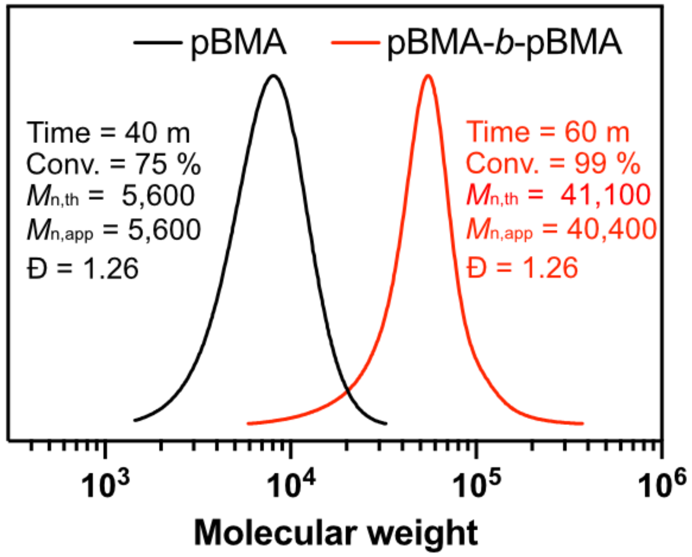

Figure S14. Chain extension of pBMA macro-initiator with BMA monomer.

Synthesis of pBMA with varying DP_T

The target degree of polymerization (DP_T = 50, 100, 200, and 400) was varied by adjusting the EBPA initiator concentration ([EBPA] = 25.2, 12.6, 6.3, and 3.15 mM) while keeping the other components at the same concentration: BMA (1.26 M), MB^+ (15.7 μM), $CuBr_2$/TPMA (0.63 mM), and TEOA (3.77 mM). ATRP "cocktail" mixture was prepared based on the general procedure and transferred to a one-dram vial (diameter = 15 mm) equipped with a magnetic stirring bar. The polymerization mixtures were irradiated for 40 min under NIR LEDs (740 nm, 20 mW cm^{-2}) under stirring (500 rpm) in open air. At the end of the polymerizations, all samples were withdrawn for gravimetric analysis and SEC measurement.

Table S1. Polymerization of BMA with varying degrees of polymerization.[a]

Entry	DP_T	[EBPA] (mM)	Conv.(%)[b]	$M_{n,th}$	$M_{n,app}$[c]	$Đ$[c]	$M_{n,abs}$[d]	Z_{avg} (nm)
1	50	25.16	75	5,600	5,600	1.26	6,300	78±0.5
2	100	12.58	99	14,300	13,700	1.17	16,100	71±0.2
3	200	6.29	91	26,000	23,500	1.19	28,300	79±0.4
4	400	3.15	83	47,400	44,600	1.24	55,100	79±0.5

[a]Reaction conditions: [BMA]/[EBPA]/[MB^+]/[$CuBr_2$/TPMA]/[TEOA] = 200/X/0.025/0.1/0.1/0.6, [EBPA] = 3.15–25.16 mM, BMA = 20 vol% to total, [HD] =10.8 wt % to BMA, [SDS] = 9.2 wt% relative to BMA, [NaBr] = 0.1 M, irradiated under NIR LED (740 nm, 20 mW cm^{-2}) for 40 min in a one-dram vial (diameter = 15 mm) in open air under stirring (500 rpm).

Temporal control under NIR light

The miniemulsion ATRP "cocktail" mixture (5 mL) was prepared based according to the general procedure for miniemulsion photoATRP at final concentrations of BMA (1.26 M), MB^+ (15.7 μM), $CuBr_2$/TPMA (0.63 mM), EBPA (6.3 mM), and TEOA (3.77 mM). The polymerization mixtures were irradiated under NIR LED (740 nm, 20 mW cm^{-2}) under stirring (500 rpm). The light was switched on/off periodically every 10 min and 100 μL of samples were withdrawn at each time point for gravimetric analysis and SEC measurement.

Table S2. Temporal control over photoATRP of BMA under NIR light.[a]

Entry	Time (min)	Light	Conv. (%)
1	0	OFF	0
2	0-10	ON	4
3	10-20	OFF	4
4	20-30	ON	25
5	30-40	OFF	27
6	40-50	ON	47
7	50-60	OFF	47
8	60-70	ON	67
9	70-80	OFF	68

[a]Reaction conditions: [BMA]/[EBPA]/[MB^+]/[$CuBr_2$-TPMA]/[TEOA] = 200/1/0.025/0.1/0.6, [M] = 20 vol% to total, [HD] =10.8 wt % to BMA, [SDS] = 9.2 wt% relative to BMA, [NaBr] = 0.1 M, NIR LEDs (740 nm, 20 mW cm^{-2}) turned on/off periodically for every 10 min in a one-dram vial (diameter = 15 mm) in open air under stirring (500 rpm).

Temporal control under red light

The miniemulsion ATRP "cocktail" mixture (5 mL) was prepared based according to the general procedure for MB^+/Cu-catalyzed photoATRP at final concentrations of BMA (1.26 M), MB^+ (15.7 μM), $CuBr_2$/TPMA (0.63 mM), EBPA (6.3 mM), and TEOA (3.77 mM). The polymerization mixtures were irradiated under red LED (640 nm, 25 mW cm^{-2}) under stirring (500 rpm). The light was switched on/off periodically every 15 min and 100 μL of samples were withdrawn at each time point for gravimetric analysis and SEC measurement.

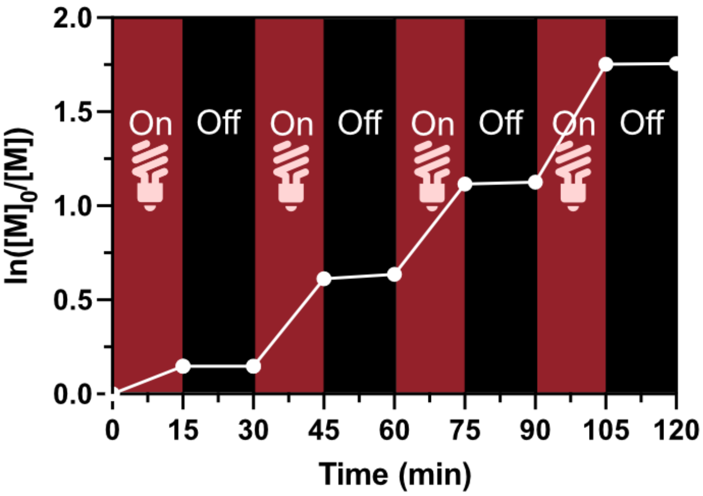

Figure S15. Temporal control for miniemulsion photoATRP under red light.

Table S3. Temporal control over photoATRP of BMA under red light.[a]

Entry	Time (min)	Light	Conv. (%)
1	0	OFF	0
2	0-15	ON	11
3	15-30	OFF	11
4	30-45	ON	43
5	45-60	OFF	44
6	60-75	ON	64
7	75-90	OFF	65
8	90-105	ON	80
9	105-120	OFF	80

[a]Reaction conditions: [BMA]/[EBPA]/[MB^+]/[$CuBr_2$-TPMA]/[TEOA] = 200/1/0.025/0.1/0.6, [M] = 20 vol% to total, [HD] =10.8 wt % to BMA, [SDS] = 9.2 wt% relative to BMA, [NaBr] = 0.1 M, red LED (640 nm, 25 mW cm^{-2}) turned on/off periodically for every 15 min in a one-dram vial (diameter = 15 mm) in open air under stirring (500 rpm).

Miniemulsion photoATRP in reactors with different diameters under different light wavelengths

The miniemulsion ATRP "cocktail" mixture (final volume of 50 mL) was prepared based according to the general procedure for MB^+/Cu-catalyzed miniemulsion photoATRP at final concentrations of BMA (1.26 M), MB^+ (15.7 μM), $CuBr_2$/TPMA complex (0.63 mM), EBPA (6.3 mM), and TEOA (3.77 mM). The polymerization mixture was transferred to one-dram vials with different diameters (7.5 mm (A), 15 mm (B), and 27 mm (C)) as shown in Figure 4A. The one-dram vials filled with polymerization mixtures at a same height were irradiated under a different light (UV light: 390 nm, 25 mW cm^{-2}, green light: 520 nm, 25 mW cm^{-2}, red light: 640 nm, 25 mW cm^{-2}, and NIR light: 740 nm, 20 mW cm^{-2}) under stirring (500 rpm) in open air. At different time intervals, samples were withdrawn for gravimetric analysis and SEC measurement.

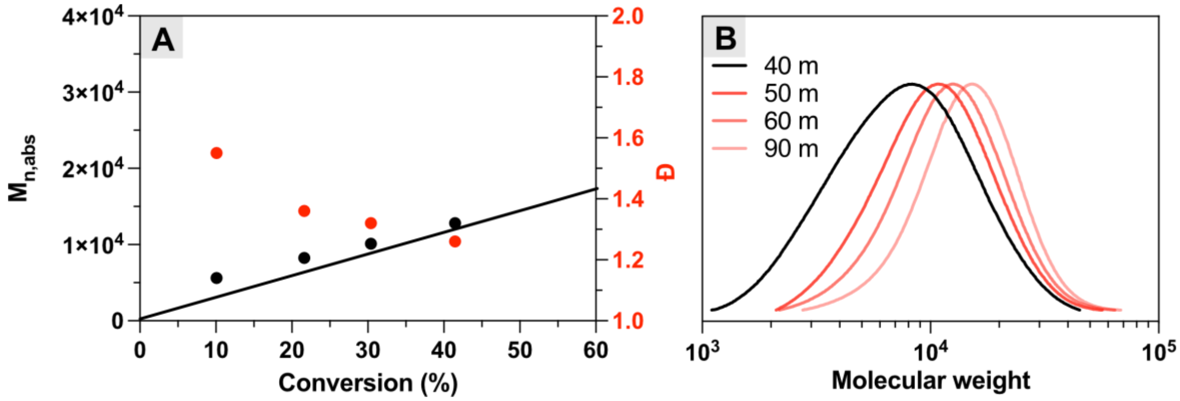

Figure S16. Kinetic analysis of miniemulsion photoATRP in a small-scale vial (diameter = 7.5 mm) under UV light. (A) Evolution of molecular weight and molecular weight distribution with monomer conversion; and (B) SEC traces evolution with time.

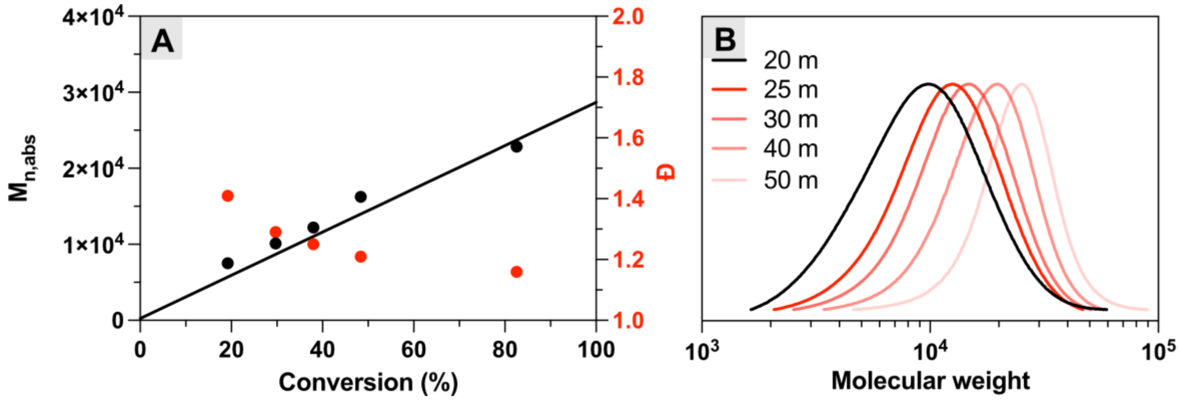

Figure S17. Kinetic analysis of miniemulsion photoATRP in a small-scale vial (diameter = 7.5 mm) under green light. (A) Evolution of molecular weight and molecular weight distribution with monomer conversion; and (B) SEC traces evolution over time.

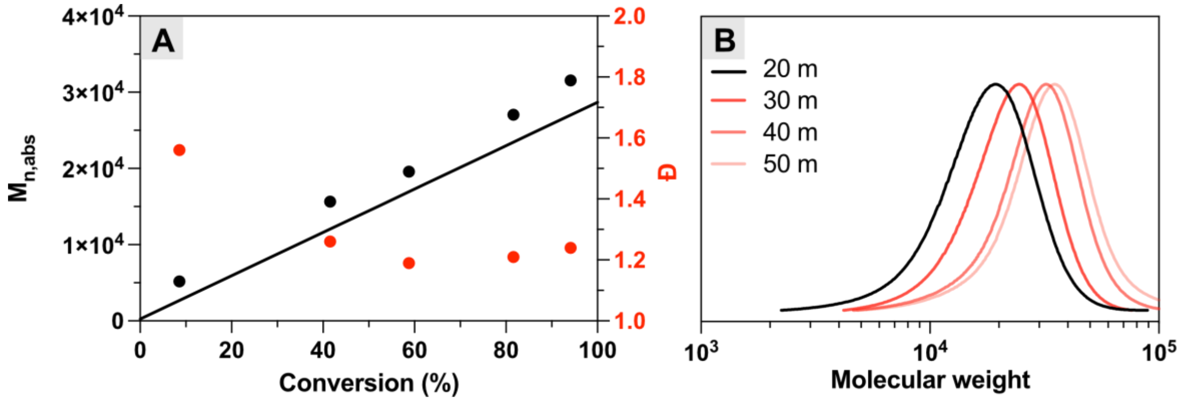

Figure S18. Kinetic analysis of miniemulsion photoATRP in a small-scale vial (diameter = 7.5 mm) under red light. (A) Evolution of molecular weight and molecular weight distribution with monomer conversion; and (B) SEC traces evolution over time.

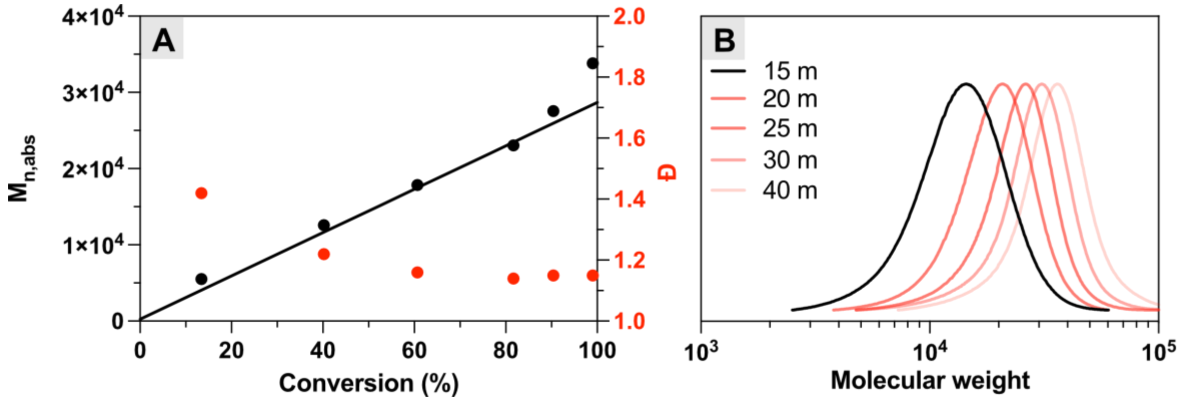

Figure S19. Kinetic analysis of miniemulsion photoATRP in a small-scale vial (diameter = 7.5 mm) under NIR light. (A) Evolution of molecular weight and molecular weight distribution with monomer conversion; and (B) SEC traces evolution over time.

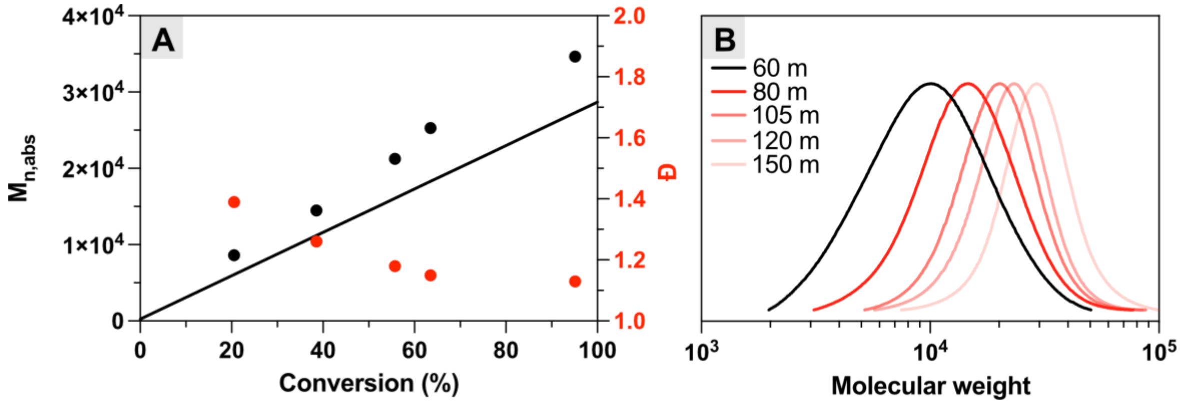

Figure S20. Kinetic analysis of miniemulsion photoATRP in a large-scale vial (diameter = 27 mm) under green light. (A) Evolution of molecular weight and molecular weight distribution with monomer conversion; and (B) SEC traces evolution over time.

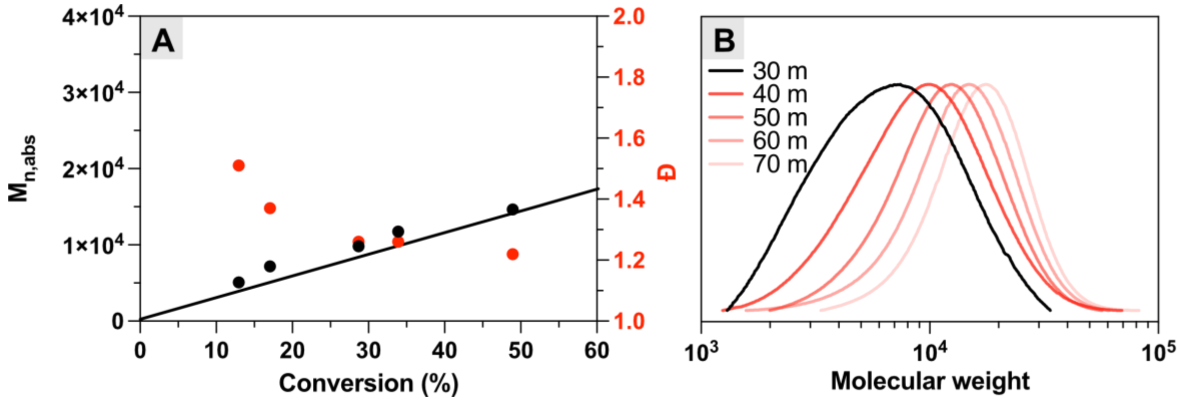

Figure S21. Kinetic analysis of miniemulsion photoATRP in a large-scale vial (diameter = 27 mm) under red light. (A) Evolution of molecular weight and molecular weight distribution with monomer conversion; and (B) SEC traces evolution over time.

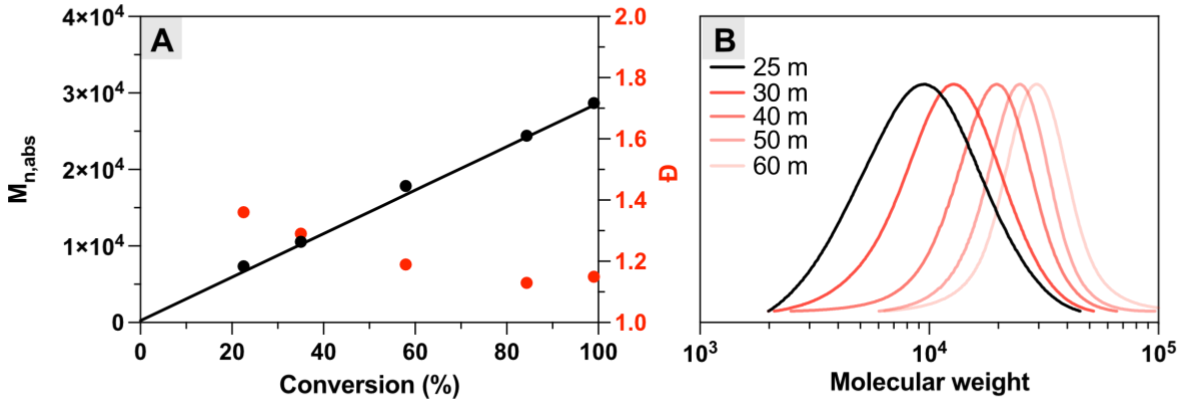

Figure S22. Kinetic analysis of miniemulsion photoATRP in a large-scale vial (diameter = 27 mm) under NIR light. (A) Evolution of molecular weight and molecular weight distribution with monomer conversion; and (B) SEC traces evolution over time.

Table S4. Miniemulsion photoATRP of BMA using NIR light in one-dram vials with varying diameters.[a]

Entry	Reactor	Time (m)	Conv.[b] (%)	$M_{n,th}$	$M_{n,app}$[c]	$M_{n,abs}$[d]	Đ[c]	Z_{avg} (nm)[e]
1	Small	40	>99	28,400	27,900	33,800	1.15	85±0.2
2	Medium	50	>99	28,400	26,100	31,600	1.19	79±0.2
3	Large	60	96	27,500	23,800	28,700	1.15	87±0.1

[a]Reaction conditions: [BMA]/[EBPA]/[MB^+]/[$CuBr_2$/TPMA]/[TEOA] = 200/1/0.025/0.1/0.6, [M] = 20 vol% to total, [HD] =10.8 wt % to BMA, [SDS] = 9.2 wt% relative to BMA, [NaBr] = 0.1 M, irradiated under NIR light with stirring, in open air.

Miniemulsion photoATRP passing through an A4 paper under red and NIR light

The miniemulsion ATRP "cocktail" mixture was prepared based according to the general procedure for MB^+/Cu-catalyzed miniemulsion photoATRP at final concentrations of BMA (1.26 M), MB^+ (15.7 μM), $CuBr_2$/TPMA complex (0.63 mM), EBPA (6.3 mM), and TEOA (3.77 mM). The polymerization mixture was then transferred to a one-dram vial (diameter = 15 mm). Polymerization through a barrier was performed by wrapping the vial with an A4 paper (~ 0.10 mm), and the reactions were irradiated under different lights (red light: 640 nm, 25 mW cm^{-2}, and NIR light: 740 nm, 20 mW cm^{-2}) under stirring (500 rpm) in open air. At different time intervals, samples were withdrawn for gravimetric analysis and SEC measurement.

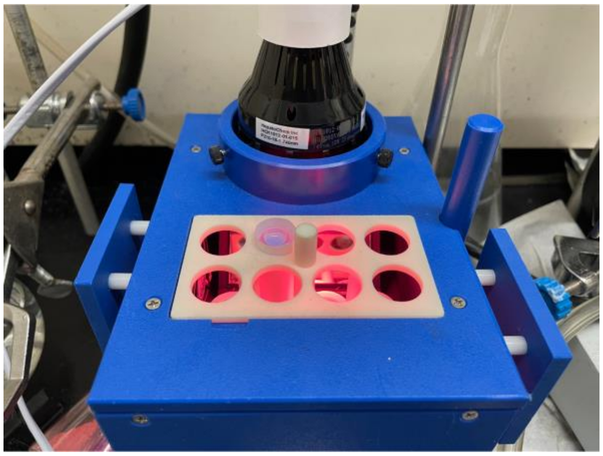

Figure S23. Experimental setup for the miniemulsion photoATRP passing through an A4 paper under NIR light.

Table S5. Comparison of miniemulsion photoATRP between without a barrier and through an A4 paper.[a]

Entry	Time (h)	Light	Conversion (%)		Conversion drop after passing through an A4 paper
			Without an A4 paper	With an A4 paper	
1	1	NIR	>99	59	40%
2	1	Red	89	33	63%

[a]Reaction conditions: $[BMA]/[EBPA]/[MB^+]/[CuBr_2/TPMA]/[TEOA]$ = 200/1/0.025/0.1/0.6, [M] = 20 vol% to total, [HD] =10.8 wt % to BMA, [SDS] = 9.2 wt% relative to BMA, [NaBr] = 0.1 M, irradiated under different light wavelengths: NIR LED (λ_{max} = 740 nm, 20 mW cm^{-2}) and red LED (λ_{max} = 640 nm, 25 mW cm^{-2}), in a one-dram vial (diameter = 15 mm) without a barrier or wrapped with an A4 paper, with stirring, in open air.

Miniemulsion photoATRP in a large scale (250 mL)

The miniemulsion ATRP "cocktail" mixture (total volume of 250 mL) was prepared based according to the general procedure for MB^+/Cu-catalyzed miniemulsion photoATRP at final concentrations of BMA (1.26 M), MB^+ (15.7 μM), $CuBr_2$/TPMA complex (0.63 mM), EBPA (6.3 mM), and TEOA (3.77 mM). The polymerization mixture was transferred to a round bottom flask (250 mL) and capped with a rubber septum as shown in Figure S23. The round bottom flask filled with polymerization mixtures were irradiated under NIR lights (740 nm, 20 mW cm^{-2}) under stirring (1000 rpm). At different time intervals, samples were withdrawn for gravimetric analysis and SEC measurement.

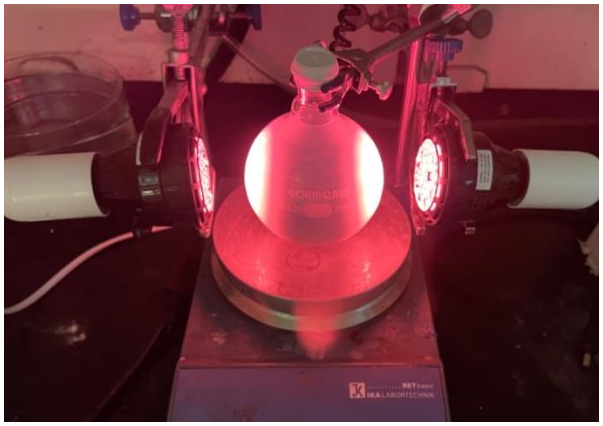

Figure S24. Photoreaction setup for miniemulsion ATRP in a large scale (250 mL) at a round bottom flask.

Table S6. NIR-light-driven ATRP miniemulsion polymerization in large scale

Entry	Reaction volume (mL)	Time (h)	Conv.[b] (%)	$M_{n,th}$	$M_{n,app}$[c]	$M_{n,abs}$[d]	Đ[c]	Z_{avg} (nm)[e]
1	250	2	60	17,300	16,000	18,900	1.23	109±0.16
2	250	3	> 99	28,400	25,500	30,800	1.26	107±0.12

Reaction conditions: [BMA]/[EBPA]/[MB^+]/[$CuBr_2$/TPMA]/[TEOA] = 200/1/0.025/0.1/0.6, [M] = 20 vol% to total, V_{total} = 250 mL, [HD] = 10.8 wt % to BMA, [SDS] = 9.2 wt% relative to BMA, [NaBr] = 0.1 M, irradiated under NIR LED (740 nm, 20 mW cm^{-2}) in a round bottom flask (250 mL) with stirring.

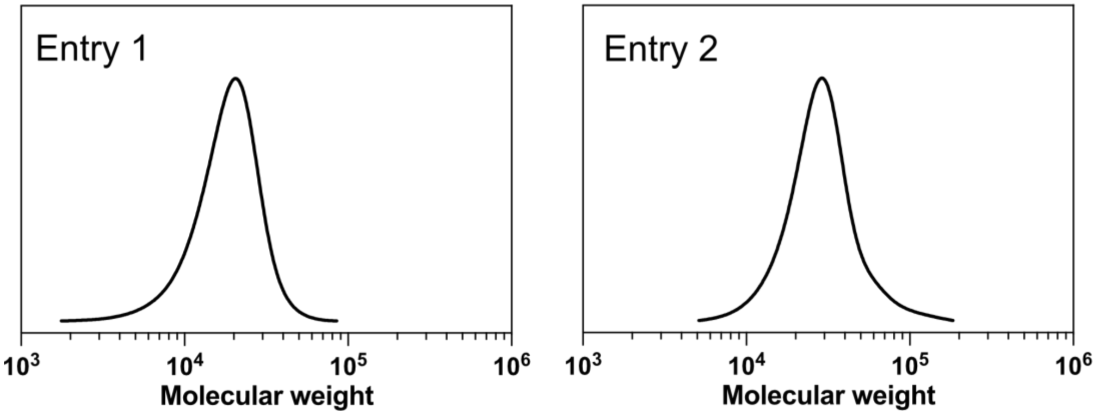

Figure S25. SEC traces of pBMAs synthesized in a large-scale reaction (Table S5)

References

1. Lorandi, F.; Wang, Y.; Fantin, M.; Matyjaszewski, K., Ab Initio Emulsion Atom-Transfer Radical Polymerization. *Angew. Chem. Int. Ed.* **2018,** *57* (27), 8270-8274.
2. Spatorico, A. L.; Coulter, B., Molecular weight determinations by gel-permeation chromatography and viscometry. *J. Polym. Sci., Polym. Phys. Ed.* **1973,** *11* (6), 1139-1150.
3. Gruendling, T.; Junkers, T.; Guilhaus, M.; Barner-Kowollik, C., Mark–Houwink Parameters for the Universal Calibration of Acrylate, Methacrylate and Vinyl Acetate Polymers Determined by Online Size-Exclusion Chromatography—Mass Spectrometry. *Macromol. Chem. Phys.* **2010,** *211* (5), 520-528.
4. Hu, X.; Szczepaniak, G.; Lewandowska-Andralojc, A.; Jeong, J.; Li, B.; Murata, H.; Yin, R.; Jazani, A. M.; Das, S. R.; Matyjaszewski, K., Red-Light-Driven Atom Transfer Radical Polymerization for High-Throughput Polymer Synthesis in Open Air. *J. Am. Chem. Soc.* **2023,** *145* (44), 24315-24327.
